# Amyotrophic Lateral Sclerosis Genes in *Drosophila melanogaster*

**DOI:** 10.3390/ijms22020904

**Published:** 2021-01-18

**Authors:** Sophie Layalle, Laetitia They, Sarah Ourghani, Cédric Raoul, Laurent Soustelle

**Affiliations:** 1 The Neuroscience Institute of Montpellier, INSERM, University of Montpellier, 34091 Montpellier, France; sophie.layalle@inserm.fr (S.L.); laetitia.they@inserm.fr (L.T.); sarah.ourghani@inserm.fr (S.O.); 2 Laboratory of Neurobiology, Kazan Federal University, 420008 Kazan, Russia

**Keywords:** amyotrophic lateral sclerosis, *Drosophila melanogaster*, SOD1, C9orf72, FUS, TDP-43

## Abstract

Amyotrophic lateral sclerosis (ALS) is a devastating adult-onset neurodegenerative disease characterized by the progressive degeneration of upper and lower motoneurons. Most ALS cases are sporadic but approximately 10% of ALS cases are due to inherited mutations in identified genes. ALS-causing mutations were identified in over 30 genes with *superoxide dismutase-1* (*SOD1*), *chromosome 9 open reading frame 72 (C9orf72), fused in sarcoma* (*FUS*), and *TAR DNA-binding protein* (*TARDBP*, encoding TDP-43) being the most frequent. In the last few decades, *Drosophila melanogaster* emerged as a versatile model for studying neurodegenerative diseases, including ALS. In this review, we describe the different *Drosophila* ALS models that have been successfully used to decipher the cellular and molecular pathways associated with SOD1, C9orf72, FUS, and TDP-43. The study of the known fruit fly orthologs of these ALS-related genes yielded significant insights into cellular mechanisms and physiological functions. Moreover, genetic screening in tissue-specific gain-of-function mutants that mimic ALS-associated phenotypes identified disease-modifying genes. Here, we propose a comprehensive review on the *Drosophila* research focused on four ALS-linked genes that has revealed novel pathogenic mechanisms and identified potential therapeutic targets for future therapy.

## 1. Introduction

ALS (amyotrophic lateral sclerosis) also known as Charcot’s disease or Lou Gehrig’s disease is a fatal adult-onset neurodegenerative disease affecting the motor system [1,2,3,4]. ALS is the most common motoneuron disorder with an incidence of two per 100,000 individuals, which varies according to geographical differences, and a mean onset at 65 [5,6]. ALS is characterized by the progressive dismantling of the neuromuscular junctions and degeneration of motoneurons in the brain and spinal cord [7,8]. Motoneuron loss leads to progressive paralysis and death due to respiratory failure within 3 to 5 years of onset disease [9]. In addition to motoneuron degeneration, ALS is clearly a non-cell-autonomous disease as astrocytes, oligodendrocytes, microglial cells, and blood-derived immune cells also contribute to the selective degeneration of motoneurons [10]. Moreover, ALS forms a broad neurodegenerative disease continuum with frontotemporal dementia (FTD) disease, and up to 50% of ALS patients concomitantly develop cognitive impairment or behavioral changes [11,12,13]. Despite recent promising gene therapy approaches, no effective cure is currently available for ALS patients [14]. Most cases of ALS are sporadic (sALS), and up to 10% have been classified as familial ALS (fALS) [15]. Currently, fALS-associated mutations have been found in approximately 50 genes, and more than 30 are thought to be causatives [16,17]. The most commonly mutated ALS-linked genes are *superoxide dismutase-1* (*SOD1*), *chromosome 9 open reading frame 72 (C9orf72)*, *fused in sarcoma* (*FUS*), and *TAR DNA-binding protein* (*TARDBP*) [18].

The antioxidant enzyme SOD1 was the first gene linked to fALS in 1993 [19]. This gene encodes a Cu/Zn superoxide dismutase, whose function is to catalyze the conversion of the superoxide ion, a toxic reactive oxygen species (ROS) produced during cellular respiration, to dioxides [20]. In 2011, abnormal GGGGCC hexanucleotide repeat expansion (HRE) within the *C9orf72* gene was identified as a new cause of ALS and frontotemporal dementia (FTD) [21,22]. Currently, intronic HRE in the *C9orf72* gene represents the most common genetic cause of ALS [23]. C9orf72 is part of a guanine nucleotide exchange factor complex [24], whose precise function remains unclear, but which was shown to be an important regulator of membrane trafficking and autophagy [25]. Lastly, *FUS* and *TARDBP* encode two DNA/RNA-binding proteins, which play distinct roles in transcription, as well as numerous roles in RNA metabolism, including splicing, stability, and transport [26]. FUS is a protein belonging to the heterogenous nuclear ribonucleoproteins (hnRNPs) (also known as hnRNP P2) [27], belonging to the FET protein family that includes two other RNA-binding proteins (RBPs) EWS and TAF15 [28]. In 2009, FUS was identified to be involved in fALS cases [29,30]. *TARDBP* encodes the TDP-43 protein, which is mainly nuclear and shuttles between the nucleus and the cytoplasm. Nuclear depletion and cytoplasmic aggregation of TDP-43 are found in most if not all ALS patients independently of the mutated status of TDP-43, making it a hallmark of the disease [31]. However, it is still debated whether TDP-43 cytoplasmic aggregation is deleterious or protective for ALS disease [32].

**Figure 1:**
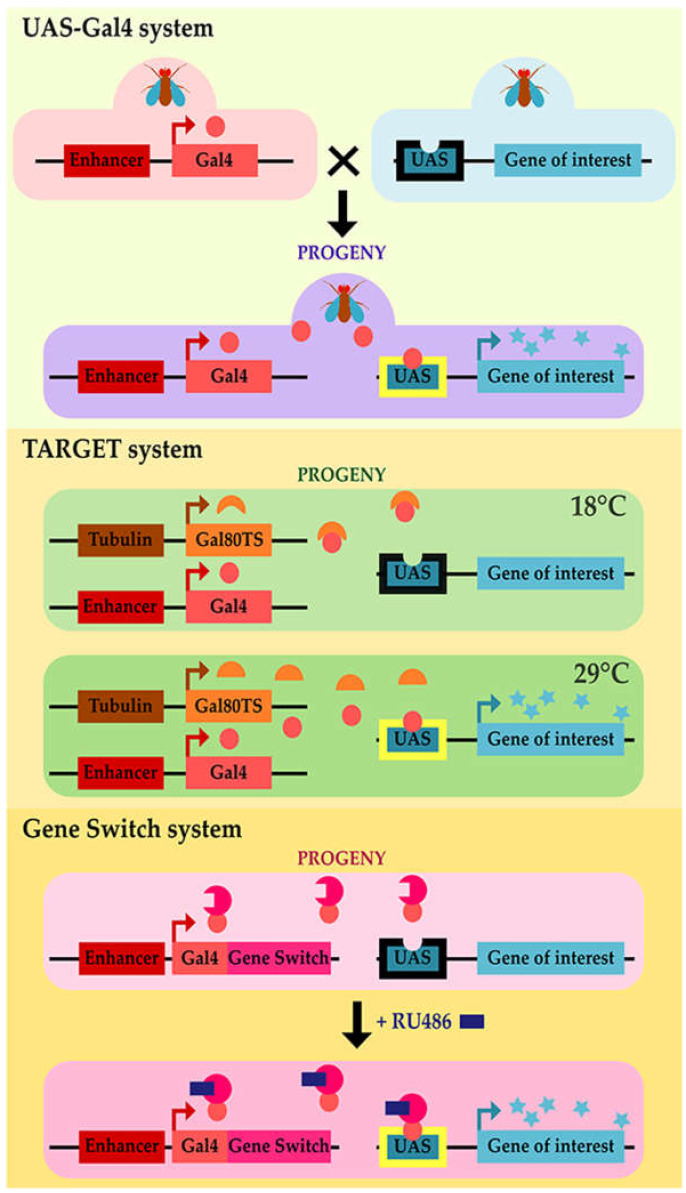
*Drosophila* genetic tools. The Gal4 system introduced in *Drosophila* allows transgene ectopic expression with spatiotemporal control. The upstream activating sequence (UAS)/Gal4 is a bipartite system. One transgenic line expresses the Gal4 transcription factor from yeast in a tissue-specific manner using an endogenous promoter or an enhancer sequence. The other line carries the transgene under the control of the UAS (upstream activating sequence). In the progeny of the cross, the UAS is bound by Gal4 protein and the transcription of the gene of interest starts. The temporal and regional gene expression targeting (TARGET) method allows a more flexible temporal control of the Gal4 system. A temperature-sensitive version of Gal80 protein (Gal80TS) is expressed ubiquitously under the control of the Tubulin promoter. At permissive temperature (18 °C), Gal80TS bound to Gal4 and prevents the starting of the transcription. A temperature shift to 29 °C relieves the transcriptional repression and permits the transcription of the gene of interest. The GeneSwitch system is based on hormone-inducible Gal4 and allows a temporal regulation of transgene expression. Gal4 is fused to a progesterone receptor, and the addition of hormone or synthetic ligand (RU486) in the feeding of the fly (adults or larvae) activates the transcription of the gene of interest. This system is reversible as the removal of ligand silent the system. All these genetic tools are routinely used in laboratory to precisely control transgenes expression not only in a specific tissue but also in a precise temporal manner.

**Figure 2:**
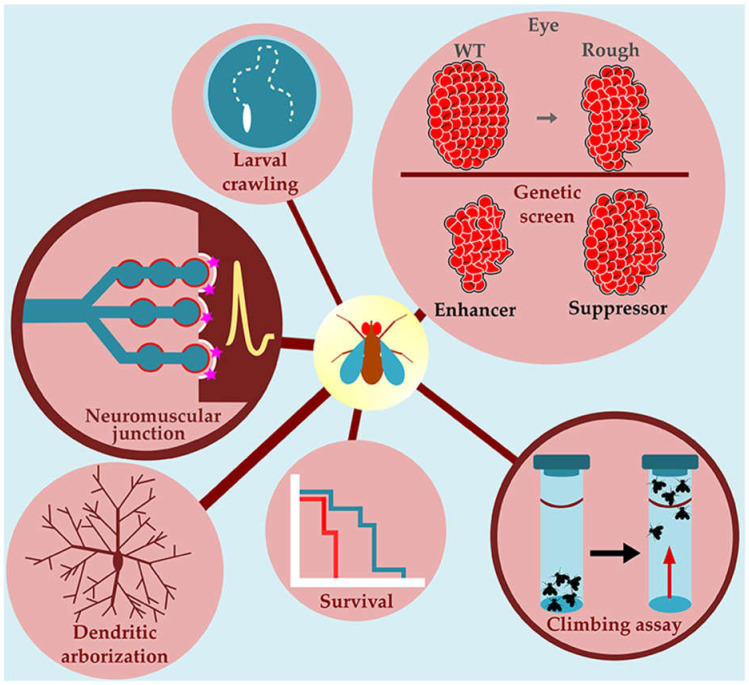
*Drosophila* is a model to study neurodegenerative diseases. Several neurodegenerative diseases, including amyotrophic lateral sclerosis (ALS), have been modeled in *Drosophila* via transgenic expression of wildtype or mutated human proteins. The toolkit available at *Drosophila* allows an in-depth study of the neurodegenerative mechanisms associated with these diseases. Several behavioral tasks, such as larval crawling and climbing assays, allow monitoring the locomotor activity during *Drosophila* life. *Drosophila* lifespan assays are useful to follow the time course of neurodegeneration and might be used as a readout for genetic screens. When expressed in the eye, toxic proteins disrupt the stereotyped organization of ommatidia and bristles, leading to a rough eye phenotype. This easily observable readout allows to perform genetic screens aimed at identifying modifiers (enhancers or suppressors) of the rough eye phenotype. Dendritic arborization neurons have a complex dendritic branching pattern and are widely used to identify genes involved in dendrite morphogenesis processes. The larval neuromuscular junctions (NMJs) are glutamatergic synapses that use ionotropic glutamate receptors and post-synaptic scaffolding proteins sharing similarities with mammalian brain synapses. These NMJs are easy to visualize and relatively simple with few axonal branches composed of synaptic boutons, which contain the active zones (sites of neurotransmitter release). Morphological and electrophysiological analyses on larval NMJs are frequently used to study how gene loss or gain of function might influence synapse development and function.

*Drosophila melanogaster* is a model easy to handle, cost-effective, with a short lifespan and a fully sequenced genome since 2000 [33,34]. In addition, *Drosophila* is a powerful genetic model with several genetic tools, such as the upstream activating sequence (UAS)/Gal4 system (Figure 1) [35], which is extensively used to overexpress *Drosophila* or disease-associated human genes in a tissue/cell-specific manner. Combined with the temporal and regional gene expression targeting (TARGET) or gene-switch systems [36] (Figure 1), gene expression can be controlled temporally allowing to investigate behavioral studies, avoiding developmental alterations. Furthermore, pan-genomic screenings, using RNA interference (RNAi)-induced gene knockdown for example, have been successfully used to identify genetic modifiers of human disease-associated phenotypes. It is estimated that as many as 77% of the human disease-associated genes have fly orthologs [37]. Furthermore, 76% of human proteins involved in synaptic vesicle trafficking have a *Drosophila* ortholog [38], indicating that synaptic transmission machinery is well conserved in flies. For all these reasons, *Drosophila* has emerged as a powerful genetic model for studying several neurodegenerative diseases (for reviews, see [39,40,41]) (Figure 2). Genetic studies in *Drosophila* have provided novel insights into the cellular and molecular mechanisms of ALS-linked neurodegeneration. Here, we review the *Drosophila* models that have been developed to better understand the function and decipher the pathological consequences associated with *SOD1*, *C9orf72*, *FUS*, and *TARDBP* genes.

## 2. *SOD1* Gene

### 2.1. dSod1 and the Aging Theory

In the 1980s, the concept of the aging process emerged with the assumption that aging reflects over time an accumulation of changes associated with an increasing susceptibility to develop diseases and, finally, death [42]. The free-radical theory assumed that the basic cause of aging lies on the deleterious effects produced by free-radical reactions [43,44,45,46]. This theory was supported by the observation that caloric restriction or lowering the metabolic rate decreases free-radical production, increasing the average lifespan in different species including *Drosophila* [47]. The prediction was to extend lifespan by increasing antioxidant levels to overcome the damages caused by free-radical reactions. As the main source of free radicals in aerobic eukaryotes is generated by oxygen metabolism, in that context, many studies have focused on the *dSod1* gene. At that time, the link between ALS disease and the mutations in *hSOD1* was not known, and the idea was to genetically increase lifespan in animal models especially in *Drosophila* using *dSod1*.

In *Drosophila*, the gene *dSod1* was cloned in 1989 [48]. Many studies have shown that different genetic conditions leading to *dSod1*-null mutants, such as deletions or missense mutations inactivating the enzymatic activity of the protein, exhibited several phenotypes. The lifespan was drastically reduced by 85–90% and the locomotor activity was also impaired. The resistance to oxidative stress conditions was lowered in *dSod1*-null animals, whereas abnormal wing morphology and infertility were also observed [49,50,51]. The *dSod1* loss-of-function (LOF) phenotypes were unsurprising for a ubiquitous housekeeping enzyme involved in detoxification and remained in the line with predictions and aging model. More unexpected were the consequences of *dSod1* gain of function (GOF). Transgenic *Drosophila* lines carrying an additional copy of *dSod1* were constructed using random P element insertion [52]. These flies showed 30–40% increase in the dismutase activity but with a minor effect on both lifespan and oxidative stress resistance (Table 1). One explanation was that the increase in dSod1 activity level was too low to markedly increase the maximal lifespan of these flies. On the other hand, a previous study using transgenic flies expressing the bovine form of SOD1 under the control of actin5C promoter showed that ubiquitously increasing bovine SOD1 expression to high levels led to a deleterious effect with flies that did not emerge from their pupal cases [53].

### 2.2. Drosophila as a Modeling Tool to Understand hSOD1-Induced ALS

#### 2.2.1. hSOD1 Was the First Gene Linked to ALS Disease

**Figure 3:**
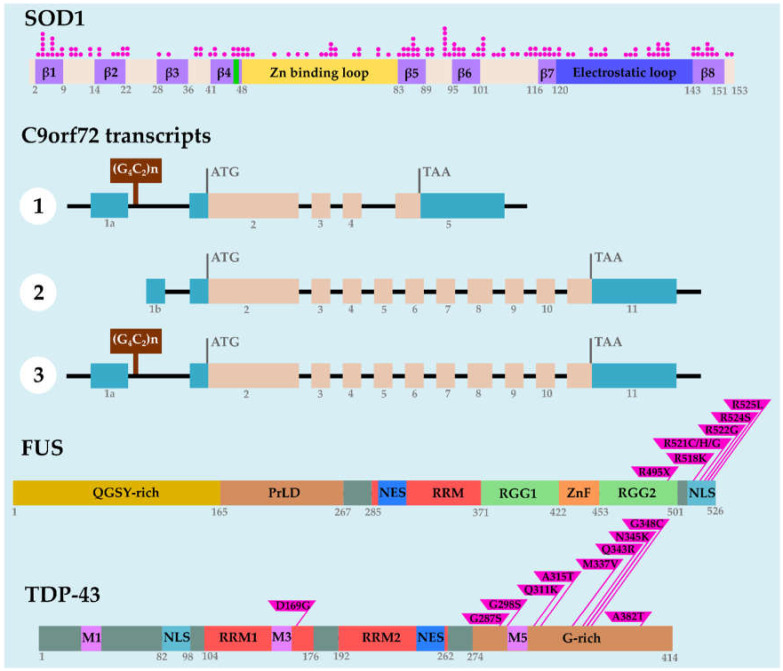
Structure of chromosome 9 open reading frame 72 (C9orf72) transcripts and SOD1, fused in sarcoma (FUS), and TAR DNA-binding protein (TDP)-43 proteins. The human SOD1 protein is composed of eight beta-strands (in purple) and connecting loops. The Cu-binding region, the Zn-binding loop, and the electrostatic loop are represented in green, yellow, and blue, respectively. ALS-associated missense mutations are located throughout the protein and are listed vertically in pink dots. The human *C9orf72* gene produces three variants. The (G_4_C_2_) hexanucleotide repeat expansion (HRE) is located in the first intron of variants 1 and 3, and in the promoter region of variant 2. The human FUS protein constitutes a N-terminal glutamine/glycine/serine/tyrosine-rich region (QGSY-rich in dark yellow), a prion-like domain (PrLD in brown), a nuclear export signal (NES in blue), a RNA recognition motif (RRM in red), two arginine/glycine-rich regions (RGG1 and RGG2 in green), a zinc finger domain (ZnF in orange), and a C-terminal non classical nuclear localization signal (NLS in cyan). Most ALS-related mutations are found in the C-terminal NLS region of FUS protein. The human TDP-43 protein carries three mitochondrial localization domains (M1, M3, and M5 in pink), a nuclear localization signal (NLS), two RNA recognition motifs (RRM1 and RRM2 in red), a nuclear export signal (NES), and a glycine-rich domain (G-rich in brown). The ALS-associated mutations described in the review are indicated.

In 1993, the discovery of the genetic linkage between *hSOD1* and the fALS [19] changed not only the vision of the disease but also the way the pathogenicity of *SOD1* gene was considered. Indeed, to date, about 200 ALS-associated mutations in *hSOD1* have been described [59]; most of them are missense point mutations (Figure 3). The first lines of research considered to explain the death of motoneurons characteristic in ALS were oriented toward an LOF hypothesis. The mutated form of hSOD1 protein could lead to a decrease in the superoxide dismutase enzymatic activity, implying an impairment of free-radical elimination, leading to an LOF phenotype. Nevertheless, hSOD1 mutants showed different degrees of alteration in superoxide dismutase activity. For example, hSOD1^G93A^ (at position 93, a glycine is substituted to an alanine) showed an identical enzymatic activity compared to wildtype protein [60]. In mice models expressing the G93A or G37R mutation in hSOD1, the level of dismutase activity was not altered but motoneuron degeneration still took place [8,61]. In addition, the lack of phenotype observed in *mSOD1* knockout mice [62] strengthened the idea that motoneuron death in ALS is not only from a loss in dismutase activity. Indeed, it was shown that the mutations found in *hSOD1* induce the misfolding of the protein and confer new noxious properties. This toxic GOF is worsened by the ability of misfolded hSOD1 to spread from cell to cell causing the propagation of the disease [63,64,65,66,67,68,69,70,71,72,73].

#### 2.2.2. Gain-of-Function *Drosophila* Models

As we mentioned above, *dSod1*-null mutants showed a severe reduction in lifespan. In a *dSod1*-null background (*dSod1^x16/sodx39^*), expression of *hSOD1^WT^* under the control of endogenous *cis*-regulatory *dSod1* sequences fully rescued the lifespan reduction as *dSod1* expression did. In this genetic background, the expression of different fALS-related *hSOD1* mutant alleles (*hSOD1^A4V^*, *hSOD1^G37R^*, *hSOD1^G93C^*, *hSOD1^G41D^*, *hSOD1^I113T^*) showed a partial rescue of the lifespan compared to *dSod1^x16/^*^sodx39^ flies. However, the lifespan of fALS-related *hSOD1* flies was shortened compared to flies expressing *hSOD1^WT^* allele. This lifespan reduction was coupled with an early drop of negative geotaxis performance in line with pathological phenotypes [56]. This first study did not allow the targeting of hSOD1 expression specifically in motoneurons, and the level of expression was limited to the endogenous level of *Drosophila Sod1*. *Drosophila* models were, therefore, generated using the UAS/Gal4 system [35] to overexpress different *hSOD1* transgenes (Figure 1). Targeting wildtype hSOD1 or different mutated forms found in patients directly in motoneurons allowed analyzing the consequences of this expression at the whole animal level. Using a motoneuron Gal4 driver (D42-Gal4) to express either wildtype or ALS-related forms of hSOD1 (A4V or G85R), Nancy Bonini’s team showed that these different forms did not alter *Drosophila* lifespan [74] (Table 2).

These flies displayed progressive motor function deterioration over time. Contrary to what is observed in vertebrates, no neuronal cell death has been demonstrated in the adult ventral nerve cord where the motoneuron cell bodies are located. Therefore, ALS *Drosophila* and mouse models showed different phenotypes and specificity. In mice, directing mutant hSOD1 to all neurons did not cause motoneuron disease [78,79,80], while motoneuron death is contingent on the ubiquitous expression of mutant hSOD1. In the same manner, the presence of mutant hSOD1 aggregates was not obvious in *Drosophila* nervous system compared to mouse models of ALS [68,70,81,82]. The insoluble hSOD1 inclusions cannot explain the observed motoneuron dysfunction. However, misfolded hSOD1 mutant proteins could be detected in flies using conformation-specific antibodies developed in mice [83,84]. Indeed, a study using the hSOD1^G93A^ transgene expressed under the control of the muscle specific driver (24B-Gal4) showed that misfolded hSOD1 protein was produced in muscles. This expression led to shortened lifespan, drop in motor activity, and mitochondrial impairment [77] (Table 2). Unfortunately, this study did not investigate the effects of the *hSOD1^G93A^* transgene expression in the nervous system. Interestingly, when hSOD1 mutant forms were cell-autonomously expressed by motoneurons, glial cells exhibited a stress response as evidenced by the expression of the heat-shock protein 70 (HSP70) [74]. The role of heat-shock protein in endoplasmic reticulum stress and, more particularly, the activation of the unfolded protein response (UPR) have been extensively studied in ALS disorder [85,86,87,88] (for a review, see [89]). Consistent with the important cross-talk between neurons and glial cells, recent studies suggested the idea of a non-cell-autonomous role of the UPR to modulate ALS progression [90,91,92]. The stress response induced in glial cells could, thus, help to provide support to motoneurons in the first stages of the disease. For example, HSP70 upregulation was protective during the disease progression in mouse models of ALS helping to maintain motoneuron innervation [93]. In a more general way, although the motoneurons are the main cells affected, ALS-related hSOD1 mutants in a non-cell-autonomous manner and the glial cells also play a part in ALS pathogenesis. The importance of glial cells was highlighted in BMAA (β-*N*-methylamino-l-alanine) resistance study. BMAA is a neurotoxin found in cycad seeds that causes Guam disease, an ALS–Parkinsonism dementia complex [94]. The expression of hSOD1 mutant (A4V, G85R) transgenes in either *Drosophila* motoneurons or glial cells led to different results. Expressed selectively in glial cells, the sensibility to BMAA was increased with a loss of motor performance over time. When hSOD1 constructs were expressed in motoneurons or in both motoneurons and glial cells, the resistance to the neurotoxin was enhanced, as well as the motor functions [76]. The mechanism leading to these different phenotypes according to the targeted cell type remains unknown, but it shows that glial cells are involved in the course of the disease as documented in rodent models of ALS [63,95,96].

#### 2.2.3. Knock-In hSOD1 in *Drosophila* Led to Unexpected Results

The overall literature on the study of hSOD1 ALS-associated forms has shown that the phenotypes observed were not only dependent of the transgene expression level but were also related to the cellular type targeted. To overcome these difficulties and increase the understanding of the mechanisms leading to the toxicity of the SOD protein in ALS, recent studies chose to express hSOD1 at an endogenous level. For this, they used the ends-out homologous recombination strategy to replace wildtype *dSod1* and introduce ALS-related mutations at conserved residues in *dSod1*, thereby creating *dSod1*^G85R^, *dSod1*^H71Y^, and *dSod1*^H48R^ mutants [57]. In homozygous conditions, these mutants die throughout development, with escaper adult flies showing shortened lifespan and severe locomotion defects. Although these phenotypic traits are characteristic of ALS, the different mutants showed developmental defects, as *dSod1^G85R^* homozygous died at the pharate stage and did not emerge properly. Surprisingly, despite the severe locomotor defects observed, no motoneuron death was detected in larvae or adult flies. Interestingly, all mutants produced significant amounts of dSod1 protein, and expression of wildtype dSod1 partially saved the *dSod1^G85R/G85R^* phenotypes, leading to the hypothesis that both toxic GOF and LOF are combined to explain the observed ALS-associated phenotypes. Further study of *dSod1^G85R^* mutants at the neuromuscular junction (NMJ) level showed that both the number of boutons and the neurotransmission were reduced in the mutant, at the pharate stage [58]. Earlier in development, larvae *dSod1^G85R^* displayed a clear crawling impairment that was not associated with a disturbing phenotype at the NMJ level. Electrophysiological recordings of NMJs and motoneurons did not show any difference in post-synaptic excitatory events between *dSod1^G85R^* and wildtype flies, although motoneurons exhibited a slightly reduced excitability. The crawling behavior is generated by the central pattern generator network, an innate neural circuit composed of interneurons and motoneurons that generate rhythmic motor output. Sensory inputs play a role in adapting locomotor activity to external cues. Extracellular recordings and deafferentiation experiments showed that defective sensory feedback lead to reduced locomotor activity in *dSod1^G85R^* larvae [58] (Table 1). These findings highlight previous existing hypotheses on non-cell-autonomous factors involved in ALS degeneration [95].

### 2.3. ALS Drosophila Model to Test Neuroprotective Drug Candidates

*Drosophila* studies also allowed, in the ALS context with ectopic hSOD1 expression, to easily test different compounds for their protective or negative actions, on lifespan or motor activity for example. Thus, α-lipoic acid (LA) produced from plants and known for its various properties including antioxidant potential [97] has been tested in *Drosophila* expressing hSOD1^G85R^ in motoneurons. LA was shown to moderate the neurotoxicity, extending lifespan and improving motor activity, in hSOD1^G85R^ flies [98]. In the same technical manner, γ-oryzanol (Orz), a component of rice bran oil known for its antioxidative activity was also tested [99]. In the *Drosophila* ALS context, Orz increased HSP70 expression and alleviated oxidative damage [75]. These results open the door to other studies to investigate the role of new drugs potentially neuroprotective for ALS disease.

Until now, studies concerning hSOD1 have focused on major phenotypes (longevity, motor deficit), but a more detailed analysis at the cellular level of the hSOD1 actions could provide answers on the pathways leading to the pathology. Moreover, future studies using *Drosophila* could help to understand the mode of propagation of misfolded hSOD1, as well as the relationships between different cell types (motoneurons, glial cells, muscles).

## 3. C9orf72 Repeat Expansions

In 2011, intronic GGGGCC (G_4_C_2_) HRE in the *C9orf72* gene was identified as a new cause of ALS [21,22]. In healthy individuals, the number of repeats is below 30, while it may reach thousands in ALS patients [100]. Actually, it is clearly established that abnormal HRE is the most common genetic cause of ALS [23], which accounts for around 40–50% of fALS and 5–10% of sporadic cases [23]. The human *C9orf72* gene produces three alternative spliced transcripts [21] and, depending on the splice variant, the (G_4_C_2_) repeat is present in the promoter region (for transcript variant 2) or within the first intron (for transcript variants 1 and 3) (Figure 3). To date, the underlying mechanisms of neurodegeneration associated with *C9orf72* HRE have been classified into three nonexclusive categories: (1) loss of *C9orf72* function through haploinsufficiency, (2) sequestration of proteins by HRE-induced RNA foci, and (3) toxic GOF induced by dipeptide repeat proteins.

### 3.1. Loss of C9orf72 Function

Several studies have shown that the HRE in *C9orf72* gene decreases the levels of messenger RNA (mRNA) [21,101] and protein [102,103] in patient tissues, suggesting that reduced C9orf72 protein function may play a role in the disease. *C9orf72* LOF in *Zebrafish* [104] and *Caenorhabditis.elegans* [105] induced motor deficits, but degeneration of motoneurons was not observed in mice lacking *C9orf72* gene [106]. However, a recent study showed that reduced *C9orf72* function may exacerbate the repeat-dependent gain of toxicity [107], suggesting that the loss of *C9orf72* function may be involved in the disease. The *Drosophila* genome does not contain a *C9orf72* ortholog gene [108], making it impossible to determine the consequence of *C9orf72* LOF in this organism.

### 3.2. Sequestration of Proteins by Expanded Repeat in RNA Foci

A hallmark found in different tissues, including motoneurons, of *C9orf72*-linked ALS patients is the presence of RNA foci [109]. These RNA foci are the result of bidirectional transcription of the HRE, leading to the accumulation of repeat-containing RNA aggregates, generally localized in the nucleus but also found in the cytoplasm. In *Drosophila*, it was found that components of the *Drosophila* DRB sensitivity-inducing factor (dDSIF) and *Drosophila* polymerase-associated factor 1 (dPAF1) complexes, two regulators of RNA polymerase II, are selectively required for the transcription of the expanded (G4C2) repeats [110,111]. Indeed, RNAi-induced silencing of these components decreased the RNA production only from long expanded repeats, leading to reduced toxicity. One possible toxic effect of these RNA foci is that they may sequester RBPs, interfering with their function and altering general RNA metabolism. For example, Xu et al. incubated biotinylated (G_4_C_2_)_10_ repeat RNA with mouse spinal cord lysates and identified Purα as a protein able to specifically bind to the repeat [112]. Purα is an evolutionarily conserved RBP that modulates transcription and translation, and it is also a component of ribonucleoprotein granules [113]. The authors used a UAS construct carrying a (G_4_C_2_)_30_ repeat cloned upstream of the translation start of GFP to express it in the developing eye. This induced a rough eye phenotype, revealing the toxicity of the expanded repeat that was not observed when the same construct carrying only 3 repeats was used. Overexpression of Purα with the (G_4_C_2_)_30_-GFP construct suppressed the neurodegeneration induced by the (G_4_C_2_)_30_-GFP transgene, supporting the idea that Purα function was attenuated in the presence of the expanded (G_4_C_2_) repeat. When the (G_4_C_2_)_30_-GFP construct was expressed in motoneurons using the OK371-Gal4 driver, around 50% adult eclosion failure and locomotion defect in 28 day old flies were observed. In addition, larval NMJs showed a reduction in active zone number. These phenotypes were suppressed by the overexpression of Zfp106, a zinc finger protein that binds specifically to a (G_4_C_2_)_8_ repeat construct [114]. Interestingly, Zfp106 interacts with other RBPs including TDP-43 and FUS, suggesting that its sequestration by the expanded repeat may alter the function of ALS-related RBPs.

It was shown that G_4_C_2_ HRE RNA forms hairpin and G-quadruplex structures that have the potential to recruit and sequester proteins. One such example is RanGAP (Ras-related nuclear GTPase-activating protein) that binds preferentially the sense RNA quadruplex of the HRE [115]. RanGAP stimulates the hydrolysis of GTP to GDP carried out by Ran GTPase, which is involved in nucleocytoplasmic transport. The overexpression of RanGAP suppressed the neurodegeneration induced by (G_4_C_2_)_30_-GFP expression in the developing eye. This was also the case when eye-expressing (G_4_C_2_)_30_-GFP flies were fed with 5,10,15,20-tetrakis-(*N*-methyl-4-pyridyl)porphine (TMPyP4), a porphyrin compound that destabilizes RNA G-quadruplex tertiary structures. This indicates that the G-quadruplex structure was required for the recruitment of RanGAP on (G4C2) HRE RNA [115].

Burguette et al. found that (G_4_C_2_) expanded repeat RNAs, which generally aggregate as nuclear RNA foci, also localize to neuritic granules that were actively transported along neuronal processes [116]. The presence of such hexanucleotidic repeat RNAs containing granules in neurites was associated with defects in neuronal branching, suggesting that they may confer local toxicity [116]. The class IV epidermal sensory dendritic arborization neurons of *Drosophila* have a complex dendritic branching pattern that was altered by the expression of a (G_4_C_2_)_48_ RNA repeat. The overexpression of dFMR1 or Orb2, two transport granule components that regulate local translation, enhanced the dendrite branching defects, while their RNAi-induced knockdown rescued the phenotype [116]. This suggests that local translation may be affected by the presence of (G_4_C_2_) HRE RNAs in neuronal processes.

Despite the putative sequestration of RBPs by HRE RNAs, several studies have demonstrated that RNA foci per se were not toxic. Tran et al. used a *C9orf72* minigene containing (G_4_C_2_)_160_ repeat flanked by human intronic and exonic sequences [117]. When ubiquitously expressed using the actin-Gal4 driver, they did not detect antisense RNA foci but they observed an average of 42 sense nuclear RNA foci in glutamatergic neurons and 49 in glial cells. However, adult brain transcriptome analysis revealed that transcription was quite normal, strongly suggesting that sense RNA foci were not sufficient to alter global mRNA expression.

Depending on *C9orf72* splice variant, the (G_4_C_2_) repeat is present in the promoter region or within the first intron (Figure 3). To answer whether the genomic location of the repeat could influence its toxicity, Moens et al. used two types of “RNA-only” constructs containing 100 copies of the repeat. One type of construct contained the sense or antisense repeats upstream of a polyA (sense/antisense polyA) and the other one contained the repeat within an artificial intron introduced into the GFP coding sequence (sense/antisense intronic) [118]. When expressed in adult *Drosophila* neurons, sense and antisense repeat-polyA constructs induced RNA foci within the cytoplasm, while RNA foci were mostly nuclear with both the sense and antisense intronic constructs. Despite the presence of these RNA foci, no effect on survival or climbing ability was observed indicating that they were not the primary cause of neurotoxicity [118]. However, this study did not investigate whether the co-expression of sense and antisense repeats could cause any detrimental effect.

### 3.3. Dipeptide Repeats Protein Toxicity

Despite its intronic localization, the (G_4_C_2_) expansion undergoes repeat-associated non-AUG (*RAN*) translation from both sense and antisense transcripts to produce five dipeptide proteins (DPRs) [109,119,120]. The sense transcript gives rise to poly-glycine/alanine (poly-GA) and poly-glycine/arginine (poly-GR) while the antisense transcript produces poly-proline/arginine (poly-PR) and poly-proline/alanine (poly-PA). Both sense and antisense transcripts produce poly-glycine/proline (poly-GP). To address whether these DPRs were toxic, Mizielinska et al. generated flies carrying transgene encoding “protein-only” by modifying the (G_4_C_2_) expansion with alternative codons [121]. Upon eye expression, they showed that (PR)_36_ and (GR)_36_ were toxic but not (GA)_36_ or (PA)_36_, indicating that only arginine-rich DPRs were toxic. When 100-copy DPRs were expressed, the toxic effect of arginine-rich DPRs was exacerbated but the other DPRs did not show toxicity. 

The toxicity of arginine-rich DPRs was also demonstrated by several other studies. For example, Drosophila expressing poly(PR)_50_ in glutamatergic neurons, including motoneurons, developed normally but were unable to escape out of the pupal case due to the absence of movement [122]. In contrast, flies expressing poly(GA)_50_ or poly(PA)_50_ developed normally [122]. The expression of an ATG-driven GFP-tagged poly(GR)_50_ induced eye degeneration that was not observed with ATG-driven GFP-tagged poly(GA)_50_ or poly(GP)_47_ constructs [123,124]. At larval NMJs, the expression of poly(GR)_100_ but not poly(GA)_100_ induced a decrease of presynaptic area, as well as a reduction in active zone number [125].

Several DPRs are found in ALS patients; however, it remains to be determined whether some DPRs can interact with others to modulate toxicity. Indeed, one study has shown that (GR)_80_ had a diffuse cytoplasmic localization in Drosophila salivary gland cells, while (GA)_80_ formed cytoplasmic inclusions [126]. Interestingly, when both (GR)_80_ and (GA)_80_ were co-expressed, part of (GR)_80_ formed cytoplasmic inclusions, suggesting that (GA)_80_ recruited (GR)_80_ into these inclusions. Of note, the same observation was found in induced pluripotent stem cell (*iPSC*)-derived human neurons [126]. The expression of (GR)_80_ induced cell loss at the wing margin that was partially suppressed by the co-expression of (GA)_80_. However, this protective effect of (GA)_80_ was not observed in the eye [126]. Thus, it is still unclear whether DPRs may interact with each other and modulate the toxicity of arginine-rich DPRs.

The transcellular spreading of misfolded proteins is thought to participate in the clinical progression of several neurodegenerative diseases. To answer whether DPRs have the ability to spread, mCherry-tagged DPRs were expressed in olfactory receptor neurons (ORNs) that target their axonal projections in fly brain. When 36 copies of (GA), (GR), or (PR) were used, no spreading was observed. In contrast, (GA)_100_ but not (GR)_100_ or (PR)_100_ was detected in fly brains after 3 days of expression in ORNs [127]. Accordingly, only (GA)_100_ was detected in axons and synaptic terminals of ORNs. Interestingly, the spreading was increased when a longer construct was used. It would be interesting to combine several DPRs to determine whether poly-GA may recruit other DPRs allowing them to spread between neurons.

In a way to understand how arginine-rich DPRs may cause toxicity, Lee et al. analyzed the interactome of GFP-tagged DPRs [124]. Among the identified interactors, 81 proteins were common to GFP-(PR)_50_ and GFP-(GR)_50_ and included some ALS-related RBPs, such as TDP-43 and FUS, for example, suggesting that arginine-rich DPRs may alter RNA metabolism. Moreover, these common interactors showed enrichment in proteins containing low-complexity sequence domains (LCDs), which mediate the assembly of membrane-less organelles. By using an RNAi genetic screen targeting the orthologous genes in *Drosophila*, the authors found that most of the genetic modifiers of GFP-(PR)_50_-induced toxicity were components of membraneless organelles such as nucleoli, the nuclear pore complex, and stress granules [124]. This strongly suggested that arginine-rich DPRs disturb the function of these organelles. In agreement with this, nucleocytoplasmic transport defects were also reported by other studies. A LOF genetic screen based on the modification of (G4C2)_58_-induced eye degeneration identified 18 modifiers that play a role in nucleocytoplasmic transport, suggesting that nuclear retention of mRNA may be a cause of toxicity [123]. By performing a targeted RNAi genetic screen to identify modifiers of the rough eye phenotype induced by the expression of (PR)_25_, Boeynaems et al. showed that nucleocytoplasmic transport was implicated in the pathogenic mechanism of *C9orf72* HRE [128]. Another DPR interactome study was performed on flies expressing DPRs in adult brain neurons [129]. This study identified mostly ribosomal proteins as interactors of arginine-rich DPRs. A genetic screen based on the overexpression of ribosomal proteins or translation initiation factors revealed that the eukaryotic translation initiation factor 1A (eIF1A) mitigated the lifespan defects induced by (G_4_C_2_)_36_ repeat or (GR)_100_ pan-neuronal expression. As eIF1A plays an important role in translation initiation, these data suggested that translation machinery might be affected by arginine-rich DPRs.

A recent study showed that poly(GR)_80_ expression in *Drosophila* muscles induced alteration of indirect flight muscles leading to defects in wing posture [130]. These alterations were the consequence of the entry of poly(GR)_80_ within mitochondria where they interacted with components of the mitochondrial contact site and cristae organizing system, leading to mitochondrial defects. Thus, it appears that poly(GR) may cause toxicity by interfering with mitochondrial functions, at least in muscle cells.

Altogether these studies have identified several pathways of *C9orf72*-associated toxicity that may highlight novel therapeutic targets. For example, reducing the function of components of the specific machinery required for transcription of expanded repeat RNA, such as dDSIF and dPAF1 complexes, should reduce levels of toxic expanded RNA, as well as the production of toxic DPRs [110,111]. Another strategy would be to target the G-quadruplex structures of the expanded repeat RNAs. Indeed, the destabilization of the G-quadruplex structure by TMPyP4 treatments seems to be efficient as it suppressed HRE-induced neurodegeneration in the eye [115]. Furthermore, Simone et al. identified small molecules that specifically stabilize the G-quadruplex structures [131]. When administrated in the food, these small molecules decreased DPR production and improved survival of *Drosophila* expressing (G_4_C_2_)_36_ repeats [131], confirming the therapeutic potential of this approach. Lastly, the mitochondrial alterations induced by poly(GR) expression in muscles was rescued by treatment with nigericin, a K^+^/H^+^ antiporter that rebalances mitochondrial matrix ion levels, opening the way to future potential therapeutic strategies [130].

## 4. FUS, an RNA-Binding Protein Associated with ALS

Fused in sarcoma, also known as translocated in liposarcoma (TLS), is a DNA/RNA-binding protein ubiquitously expressed. ALS-causative mutations in this RBP were discovered in 2009 [29,30]. In the nervous system, FUS is predominantly located in the nucleus and able to shuttle between the nucleus and the cytoplasm [132]. In patients, FUS is nuclear but FUS mutant forms are also found aggregated in the cytoplasm of neurons [29,30]. In ALS, the formation of protein aggregates is one hallmark of pathogenic mechanisms leading to motoneuron death, and the role of FUS in this process is important [65,133,134,135].

As an RBP, FUS has a pivotal role in many aspects of RNA metabolism and processing, including RNA splicing [136,137], transcription [138,139], nucleocytoplasmic transport [140], and translation (for a review, see [141]). FUS is a 526 amino acid protein encoded by 15 exons. The protein contains seven domains: an N-terminal region rich in glutamine, glycine, serine, and tyrosine residues (QGSY-rich domain), a prion-like domain, an RNA recognition motif (RRM), a nuclear export sequence (NES), two regions rich in arginine and glycine (RGG1 and RGG2), a zinc finger (ZnF) domain, and a C-terminal part containing a nonclassical PY-nuclear localization signal (NLS) motif [142] (Figure 3). ALS-related mutations are located preferentially in the C-terminal part affecting the NLS domain of the protein. The pathogenic FUS mutations are missense changes, and the R521H and R521C mutations are the most commonly found in patients. As a result, in patient postmortem studies, a strong labeling of FUS was observed in neuron and glial cell nuclei but also as aggregates in the cytoplasm. Only few point mutations have been identified in the sequence encoding the N-terminal part or prion-like domain of the FUS protein [29,30,143,144]. More recently, mutations in the 3′ untranslated transcribed region (UTR) of FUS have been described. Interestingly, these mutations affect the expression level of FUS rather than the cellular localization of the protein [145,146].

The mechanisms underlying the cause of neurodegeneration in FUS-ALS patients are still unknown even if numerous hypotheses have been made. Hence, mislocalization of FUS mutant forms in the cytoplasm leads to a disruption of FUS nuclear functions, such as transcription regulation or mRNA processing, and produces a LOF phenotype [134,147,148,149,150,151]. Conversely, the presence of FUS mutant forms in cytoplasmic aggregates creates a new toxic function of FUS in this compartment [144,152,153,154]. In an attempt to test these two hypotheses (LOF or new toxic GOF) to better understand the mode of action via which FUS mediates neurotoxicity, animal models for FUS-ALS have been generated. In vertebrate models, axonal degeneration and neuromuscular damages with protein aggregation in motoneuron were described as main phenotypes [148,155,156,157].

### 4.1. Drosophila Models of FUS-Related Neurodegeneration

In *Drosophila*, the first studies showed that eye-overexpression of wildtype FUS or different ALS-related mutated forms led to progressive neurodegeneration of the photoreceptors [147,158,159]. When expressed in the eyes, the phenotypes induced by the expression of wildtype FUS were a mild rough eye surface and a reduction in red pigment. The expression of different FUS mutants (R521C, 521H, 518K, R524S, or P525L) generated a more severe rough eye phenotype and even total depigmentation of the eyes (Table 3).

As ALS is characterized by the degeneration of motoneurons, the action of FUS expression in this specific neural population was considered. When expressed in motoneurons, all forms of FUS (wildtype and ALS-related) led to a deficit in locomotion at the larval stage followed by a lethality occurring at late pupal stage ([147,158] and Table 3). At the NMJ level, the synaptic endings were altered. No consensus has emerged concerning a clear morphological change in the bouton numbers following FUS expression in either wildtype or mutants [147,158,159,160,168,169]. However, the synaptic boutons exhibited less and aberrantly organized active zones [168,169]. The post-synaptic compartment of the NMJs, like the clustering of the glutamate receptors, was altered when FUS was expressed in motoneurons [168]. As a result, the synaptic transmission was severely impaired at the NMJs. Electrophysiological studies showed that the amplitude of the excitatory junctional potentials was decreased in FUS mutant expression conditions, strengthening the idea that synaptic defects appear earlier than MN degeneration [168,169].

Adult eclosion defect and late pupal lethality were also observed using a general neuronal driver as Elav-Gal4. Nevertheless, use of inducible Elav-Gal4-GS line has allowed showing that FUS mutant forms induced a drastic climbing decline associated with a reduced lifespan, more severe than wildtype FUS did [158]. Thus, the FUS fly models recapitulate several characteristics found in ALS patients. Nevertheless, depending on the studies, some discrepancies can be noted concerning the severity of different phenotypes. It appears that the expression level of the different forms of FUS is a critical element that must be taken into consideration for the analysis and comparison of the observed phenotypes. Thus, the viability and the adult eclosion rate when FUS is expressed in motoneurons can vary, as well as the severity of the induced rough eye phenotype [160,168].

### 4.2. From FUS Endogenous Functions to Toxicity

#### 4.2.1. Nuclear and Cytoplasmic Localization of a Shuttle Protein

The cellular localization of FUS has also been studied. As in ALS patients, mutated forms of FUS were both found in the nucleus and mislocalized in the cytoplasm, whereas wildtype FUS was always detected in the nucleus [29,30]. ALS-related FUS mutations are predominantly found in the C-terminal part of the protein containing the NLS domain. These mutations led to a redistribution of the FUS protein in the cytoplasm in all system models used. Nuclear import of FUS is mediated by the nuclear transport receptor transportin (also known as karyopherin-β2) and impairment of this interaction led to FUS mislocalization in the cytoplasm [144,152,164]. This observation gave rise to the hypothesis that loss of physiological function of FUS in the nucleus contributes to the ALS pathology [170,171,172].

To decipher FUS functions, studies have focused on the role of the *cabeza* (*caz*) gene. *caz* is the only ortholog of FUS in *Drosophila* [173]. The *caz^1^* mutants appeared morphologically normal but displayed an adult eclosion defect, and the resulting adult escapers showed reduced lifespan and deficit in locomotion ([159] and Table 4). Overexpression of FUS in the nervous system of *caz^1^ Drosophila* rescued the adult eclosion defect and restored both lifespan and locomotor deficits. By contrast, mutated forms of FUS (P525L and R522G) acted on the survival to adulthood of *caz^1^ Drosophila* with no effect on the lifespan and locomotion of adults [159].

The use of RNAi lines to knockdown *caz* gene expression in neurons showed that *caz* silencing did not affect lifespan but altered climbing performances ([150] and Table 5). *caz*-knockdown in the eye induced a rough phenotype due to apoptotic cells in the pupal retina. Indeed, this phenotype could be rescued by the antiapoptotic p35 expression [176]. The engineering of new *caz* null mutant and conditional alleles using homologous recombination confirmed the previous phenotypes of pupal lethality and locomotor defects [175]. These results corroborated the fundamental role of *caz* in neural development and argue in favor of the LOF hypothesis to explain the FUS-induced neurodegeneration in ALS. *caz* GOF produced the same phenotypes as FUS overexpression (Table 5). The larval locomotion was impaired with a reduced number of synaptic boutons and severe eye degeneration [160]. In addition, both Caz and FUS GOF induced apoptosis when expressed in motoneurons [150].

Both Caz and FUS are found in the nucleus in the nervous system. To address the role of FUS localization in toxicity, FUS constructs deleted for the NES domain were engineered in both wildtype FUS and ALS-related mutants. While FUS^WT^, FUS^ΔNES^, and FUS^ΔNES R518K^ localized in the nucleus, FUS^R518K^ was also found in the cytoplasm [158]. The double-mutant FUS^ΔNES R518K^ became nontoxic, suggesting that FUS mutant cytoplasmic localization is important for the toxicity to arise [158]. However, wildtype FUS deleted for its last 32 amino acids containing NLS domain (FUS^Δ32^) localized outside of the nucleus but did not exhibit any phenotype when expressed in the eye or in motoneurons. Moreover, addition of an NLS sequence in the FUS^Δ32^ construct led to a nuclear localization of the protein associated with locomotor defect and eye degeneration phenotypes [160]. These contradictory results do not allow deciphering clearly the location (nucleus or cytoplasm, or both) where FUS induces its toxicity.

Interestingly, the observation that the overexpression of wildtype FUS or mutant forms in the motoneurons led to a drastic downregulation of *cabeza* points out that FUS could autoregulate its own expression in *Drosophila* [164,168]. Moreover, mutations affecting the 3′-UTR region cause FUS overexpression and lead to ALS pathology [146], meaning that disruption of FUS autoregulation conducts to overexpression of wildtype FUS, which is sufficient to cause ALS.

These results strengthen (i) the notion of conservation between *caz* and FUS and (ii) the idea that the expression level of FUS is critical to trigger the neurodegeneration process [159,164,168]. Thus, elucidating the physiological functions of FUS with its different partners is necessary to better understand the mechanisms involved in the pathology.

#### 4.2.2. FUS Alters Mitochondrial Physiology

Mitochondria continually undergo fission and fusion processes; the breakdown of this balance is decisive in neurodegenerative diseases. Mitochondria disruption has been extensively reported from ALS patient studies [182,183] (for a review, see [184]). In motoneurons, expression of FUS^WT^ or ALS mutant FUS^P525L^ led to mitochondrial damages in transgenic flies. Compared to control condition, mitochondria were smaller, and the number of larger ones decreased. With FUS mutant overexpression conditions, the phenotype was more pronounced [163,185]. In addition, the mobility and the mitochondrial transport were reduced. Both anterograde and retrograde transports were affected; the frequency and duration of the transport interruption were increased, while the motile phase of mitochondrial transport was reduced [186]. Similar results were found using another neural model, the class IV dendritic arborization neurons (da neurons). Expression of FUS and Caz wildtype or mutant in da neurons altered the dendritic branching. FUS^P525L^ or Caz^P398L^ mutants localized in the cytoplasm and were found at the synaptic projections of the da neurons, which were altered. All forms of FUS and Caz (wildtype or mutant) impaired axonal transport of the synaptic vesicles. A decrease in the number of synaptic mitochondria in da neurons was also observed. The expression of all forms of FUS and Caz induced an increase in the frequency of calcium transients in da neurons [181]. FUS was also found associated with mitochondria, and it interacts directly with HSP60, an ATPase dependent mitochondrial chaperone [187], which is involved in the translocation of FUS to the mitochondria. Knocking down HSP60 expression via RNAi expression in motoneurons was sufficient to rescue the mitochondrial phenotypes induced by FUS overexpression [163]. The physiological role of FUS in mitochondria is still unknown but it seems that, in excess of FUS, the interaction of FUS with HSP60 promotes mitochondrial damage and toxicity.

#### 4.2.3. FUS in the Nucleus Is Associated with Nuclear Bodies

The nucleus is compartmentalized in membraneless intranuclear compartments collectively named nuclear bodies (NBs). NBs include Cajal bodies, nucleoli, nuclear speckles, and paraspeckles that are dynamic structures responding to stress and controlling gene expression. NBs are the location of RNA biogenesis and maturation, and they are involved in the assembling of ribonucleoprotein complexes or in the retention of proteins [188,189,190,191]. NBs are composed of various proteins and RNAs, including the architectural RNAs (arcRNAs) that are long noncoding RNAs (lncRNAs) used as scaffolds [191,192,193]. Interestingly, FUS was found associated with paraspeckles, and both LOF and GOF of FUS caused disruption of NBs [194]. In *Drosophila*, arcRNAs were found associated with hnRNPs. Notably, arcRNA hsrω is crucial for the formation of specific ω-speckles NBs. It also regulates the intranuclear trafficking and availability of different hnRNPs [195,196,197,198,199]. The loss of hsrω in neurons, using an RNAi-specific line, resulted in phenotypes closely related to the ones of *caz* LOF. Adults exhibited a shortened lifespan accompanied by locomotor deficit and a reduction in the number of synaptic boutons at the NMJs [179]. In addition, the subcellular localization of Caz changed and became cytoplasmic, leading to the conclusion that ω-speckles are involved in Caz compartmentalization. *caz* and *hsrω* genetically interact as the overexpression of hsrω enhanced the rough eye phenotype induced by *caz* expression [179]. The same interaction occurred between *hsrω* and FUS. Expressed in eye, *hsrω* RNAi rescued the toxicity induced by FUS. In this genetic condition, FUS was cytoplasmic as observed in the control condition, but punctate forms were detectable in the cytoplasm [179]. In this *hsrω*-knockdown background, FUS insoluble aggregates were not toxic and were found associated with Lysosome-associated membrane protein 1 (LAMP1), a marker of lysosomes that was upregulated [179,200]. Thus, misregulation of lncRNA could rescue the FUS-induced toxicity via the formation of nontoxic FUS aggregates through a mechanism that remains to be explored.

#### 4.2.4. FUS Is a Multidomain Protein: Structure and Function

As mentioned above, FUS is composed of different domains whose functions have been revisited in recent years. Previously, the C-terminal domain was the focus of most studies because most ALS mutations cluster in the NLS sequence and were known to disrupt the nuclear localization of FUS. Revisiting the FUS amino-acid sequence showed that the N-terminal part contains a prion-like domain followed by a glycine-rich and an arginine–glycine–glycine repeat sequence (RGG) (Figure 3). These three domains, which are composed of few different amino acids, are considered an LCD sequence. It was shown that the LCD of FUS is necessary for the formation of phase-separated liquid droplets or hydrogel [201,202,203,204]. To better understand the role of each FUS domain in the neurodegeneration process in vivo, systematic deletions or mutations of these domains have been generated in transgenic flies. 

In motoneurons, expression of wildtype FUS led to pupal death. Flies cannot emerge from the pupal case and the few escapers observed display a soft cuticle and unexpanded wings that are characteristics of an immature phenotype (Table 3). These phenotypes have been used as readout to determine the toxicity of the different domains of FUS. Bogaert et al. expressed in motoneurons different forms of FUS, deleted for distinct domains [161]. They analyzed the phenotypes obtained, comparing with the toxicity induced by wildtype FUS. In this screen, deletion of the Gly-rich domain, RGG1 domain, or zinc finger domain did not change the pupal death phenotype observed with wildtype FUS. This was also the case for the RRM domain, contrary to previous study reports ([165] and Table 3). The conclusion was that the alteration of all these domains by themselves is not sufficient to drive FUS toxicity. 

As already reported, mutations in the PY-NLS domain lead to mislocalization of FUS in the cytoplasm and induce a strong eye degeneration phenotype compared to wildtype FUS (Table 3). In motoneurons, expression of FUS lacking its NLS domain partially allowed flies to eclose, contrary to wildtype FUS that induced pharate lethality. These emerging flies stayed immature, indicating that FUS lacking its NLS domain still confers some toxicity [161]. Unexpectedly, FUS lacking its NLS had a cytoplasmic localization but did not induced significant phenotype when expressed in the eye. However, the co-expression of this mutant with wildtype FUS enhanced the rough eye phenotype. In these flies, wildtype FUS was localized in the cytoplasm of retinal cells as aggregates, leading to the idea that expression of the C-terminally truncated FUS could interact with wildtype FUS to form toxic aggregates in the cytoplasm [205]. Moreover, PY-NLS was recently shown to have a function in disaggregation via the nuclear import receptor (NIR) Kapβ2. Indeed, NIRs act as chaperones to mediate nuclear import but also have an additional function in disaggregation activity and preventing fibrillization of RBPs in in vitro experiments [206]. Kapβ2 and FUS genetically interact, as Kapβ2-knockdown in the eye enhanced the rough eye phenotype induced by FUS overexpression [206]. 

An in vitro study showed that the LCD of FUS is involved in the self-assembly process [203]. In motoneurons, deletion of the QSGY domain (the most N-terminal part of the LCD) led to a partial rescue of the phenotypes induced by wildtype FUS expression. The adult eclosion rate of these flies was recovered, but the emerging adults showed an immature phenotype like the FUS-expressing escapers [161]. When expressed in the eye, an LCD-mutated form of FUS displayed no neurodegeneration phenotype and abolished the phenotype generated by mutations located in the NLS domain. Indeed, the self-assembly of FUS in the cytoplasm, via the LCD, seems indispensable to induce the neurodegeneration process in ALS [205]. 

The lack of phenotype observed with the C-terminally truncated FUS mutant could be explained by some differences in the LCD of Caz and FUS proteins. Indeed, the QGSY domain is absent in Caz protein [173]. Thus, NLS-mutated Caz did not display any phenotype when expressed in motoneurons, but addition of a QGSY domain to this NLS-mutant form generated a toxic protein and severe eclosion reduction, as observed for FUS mutants [161]. Indeed, Caz protein is not sequestered in the cytoplasm because of the lack of interaction with LCD of the truncated forms of overexpressed FUS [164,205]. 

#### 4.2.5. FUS Is an RBP Found in Stress Granules

FUS is a protein prone to aggregate and, in patients, FUS is found in cytosolic aggregates. In fact, the LCD of FUS shares similarity with the yeast prion protein [207,208]. How FUS self-assembles in cytoplasmic aggregates in vivo to induce neurodegeneration is still an interesting question. The PrLD and RGG2 domains are required and act in *cis* to mediate FUS toxicity [161]. In vitro experiments showed that the FUS PrLD domain is necessary and sufficient for FUS fibrillization [154,209] and the LCD to undergo phase separation or sol–gel transition [149,201,203,210,211,212]. These domains are prone to aggregation and are involved in the biogenesis of membraneless organelles such as stress granules (SGs) [213]. In the cytoplasm, SGs are membraneless ribonucleoprotein compartments that have a dynamic nature. They assemble and increase in number through stress conditions, disassemble when stress is removed, or persist under chronic stress. SGs play a crucial role in RNA metabolism, notably in translation inhibition through phosphorylation of eukaryotic initiation factor 2α (eIF2α). Originally protective, under chronic stress, the presence of SGs could lead to pathological conditions (for reviews, see [214,215,216]). Thus, SGs could act as a seeding mechanism that result in accumulation of RBPs. 

In ALS patients, FUS co-localizes with SG markers [152], where it may induce a phase transition (liquid to solid) that reduces the SG dynamic [212,217]. In *Drosophila*, motoneuron expression of a FUS protein containing a RGG2 domain mutated in all arginines rescued the FUS-induced toxicity like the deletion of the entire RGG2 domain. Hence, RGG2 and LCD regions play a crucial role in FUS aggregation.

#### 4.2.6. FUS and Its Post-Translational Modifications (PTMs)

PTMs, which are known to regulate protein structure and function [218], influence protein aggregation in neurodegenerative diseases [219,220,221,222]. FUS can be post-translationally modified at various positions, leading to modification of its cellular localization, aggregation, and self-assembly tendency. Phosphorylation, acetylation, glycosylation, mono- and di-methylation, and ubiquitination have been described to occur at different positions along FUS protein (for extensive reviews, see [223,224]). Interestingly, FUS phosphorylation seems very labile in *Drosophila*, and hypophosphorylated forms of the protein alter FUS solubility properties, causing toxicity. Indeed, insoluble FUS corresponds to hypophosphorylated forms of the protein that mediate toxicity independently of inclusion formation [162]. Moreover, a detailed observation showed that the solubility profile of FUS depends on the cell type in which it is expressed. Neurons of the brain or photoreceptors of the retina could perform different FUS post-translational processes resulting in different phosphorylation patterns [162]. This suggests that the regulation of the PTMs may also be specific to each cell type and should be taken into account. Recently, arginine methylation has been shown to be a key regulator of FUS solubility and homeostasis [225,226,227]. Arginine methylation (notably on RGG2 neighboring the NLS) is also suspected to interfere with the binding affinity of NIR on the PY-NLS sequence [144,206]. The regulation of FUS methylation is mediated by the *Drosophila* arginine methyltransferase proteins (DARTs) [228]. More precisely, DART5 is involved in this process [164,229,230]. Overexpression of DART5 and FUS simultaneously rescued the degeneration induced by FUS in the eye [231]. Conversely, DART5 knockdown alone resulted in eye damage, and this phenotype was prevented by the depletion of hsrω. This result with others indicated that the lncRNA hsrω transcriptionally regulates DART5, which in turn regulates the arginine methylation of FUS protein. Interestingly, methylated FUS protein is eliminated via the proteasome that is known to act on protein in soluble phase. It is conceivable to speculate that FUS methylation is involved in the regulation of FUS solubility [231]. According to these results, PTMs could influence FUS-induced pathology and, in the future, could serve as therapeutic orientations.

### 4.3. Search for Suppressors of FUS-Induced Neurodegeneration

*Drosophila* has always been a system model used to search for toxicity-modifying genes. Several genetic screens have been done to understand the mechanisms leading to motoneuron neurodegeneration using either Caz or FUS genetic background. This approach has enabled finding different interacting proteins involved in distinct cellular processes, all implied in FUS-induced neurodegeneration.

#### 4.3.1. Nucleocytoplasmic Localization

In an attempt to understand the mechanisms that lead to FUS toxicity and neurodegeneration, the use of *caz*-knockdown induced phenotypes (rough eye and locomotor defect) has allowed the identification of *ter94* (ortholog of human *Valosin-containing protein*, *VCP*) [178]. Ter94 is an AAA ATPase. This protein family is implied in various cellular processes such as ubiquitin-dependent protein degradation, vesicle transfer, and nucleocytoplasmic transport [232,233,234]. Loss of *ter94* function enhanced the *caz*-knockdown phenotypes, and conversely the expression of *ter94* in eye or neurons can rescue the neurodegenerative phenotype observed in *caz*-knockdown flies [178]. Interestingly, VCP mutations were identified in fALS patients [235]. Recently, FUS was found mislocalized in the cytoplasm of human induced pluripotent stem cells (iPSCs) derived from VCP-mutant motoneurons [236,237]. Indeed, FUS cytoplasmic mislocalization under a diffuse form could be generalized in different ALS models, suggesting a broader role of FUS in the pathology.

#### 4.3.2. Transcriptional Regulation

More recently, the same strategy enabled highlighting the link of *caz* with Xrp1, a DNA-binding protein involved in gene expression regulation, chromatin remodeling, and DNA repair [238,239]. In neurons, the knockdown of Xrp1 rescued the *caz* mutant phenotypes [240]. Xrp1 expression is upregulated in a *caz* mutant background, and this genetic interaction is dependent on the Xrp1 DNA-binding domain. These observations led to the hypothesis that, in *caz* mutants, increased Xrp1 expression could induce gene expression dysregulation causing neurodegeneration [240]. No *Xrp1* ortholog has yet been found in mammals. Nevertheless, in the context of mutant FUS expression, Xrp1 downregulation rescues the ALS-induced phenotype, somehow suggesting conservation in the gene expression dysregulation mechanism leading to ALS pathology.

#### 4.3.3. Piwi-Interacting RNA (piRNA) Biogenesis

*caz* genetically interacts with different genes involved in the piRNA biogenesis such as *Piwi*, *Aubergine*, and *Argonaute3* [180]. piRNAs are small noncoding RNAs that regulate the chromatin structure [241,242,243]. They have been found in *Drosophila* brain, and Aubergine (Aub) is involved in piRNA biogenesis in neurons [244,245]. In *caz* knockdown many abnormal pre-piRNAs were found. Aub overexpression enhanced the neuronal defects induced in this genetic background and increased the cytoplasmic localization of Caz [180]. Indeed, Caz seems to have an action on piRNA processing. These results suggest that the creation of complexes containing pre-piRNAs, Aub, and Caz in the cytoplasm could contribute to neuronal degeneration and disorder. 

#### 4.3.4. Cytoplasmic Mislocalization and SGs

Pupal lethality and reduced adult eclosion rate phenotypes induced by FUS expression in neurons secreting the bursicon neuropeptide (also known as CCAP neurons) are particularly suitable for the design of genetic screens [166]. This approach was validated using *Pink* and *Parkin*, two genes already described to modify FUS toxicity. These genes act in a common pathway to target damaged mitochondria toward an autophagic pathway to control mitochondrial quality [246]. Downregulation of these genes in motoneurons improved the larval locomotion defect and rescued the CCAP phenotypes induced by FUS expression [166,186]. Candidate gene approaches to explore the role of genes involved in the nucleocytoplasmic transport have shown that nucleoporin (Nup154, a nuclear pore protein) and Exportin1 (XPO1, a nuclear transport protein) can suppress the neurodegeneration associated with FUS toxicity. Interestingly, these experiments highlighted the role of XPO1 for FUS localization into SGs even though the mechanism remains unclear [166]. 

#### 4.3.5. Hippo and c-Jun N-Terminal Kinase (JNK) Signaling Pathways

The rough eye phenotype induced by FUS expression was also used as a readout for genetic screens. Recently, this paradigm led to the identification of the Hippo pathway as a modifier of FUS-induced neurodegeneration [167]. Hippo signaling is a growth regulatory pathway and its LOF leads to cell proliferation. On the contrary, gain of Hippo signaling results in apoptosis or cell death through the activation of JNK signaling [247,248] (for reviews, see [249,250,251]). Downregulation of the Hippo pathway allowed the rescue of the FUS toxic effect in the eye. Indeed, FUS expression triggered the activation of Hippo and JNK signaling, resulting in neurodegeneration [167]. Moreover, the Hippo signaling pathway was also a modifier of the *cabeza* knockdown-induced phenotypes [177], strengthening the important role of this pathway in the pathology.

*Drosophila* has allowed gaining insight into the understanding of the mechanisms implied in FUS-induced neurodegeneration. However, many data seem contradictory and deserve to be investigated de novo in light of new data. For example, very recently, FUS was found to be a bicistronic gene coding for two proteins [252]. The FUS coding sequence contains a second protein-coding sequence in a shifted frame in the prion-like domain. The new protein of 170 amino acids (altFUS, which has its coding sequence in the 5′ region of the mRNA) seems to be involved in mitochondrial defects when overexpressed. Even if the primary results did not lead to an obvious action of altFUS in *Drosophila* [252], a more precise study to understand the physiological functions of the two proteins (FUS and altFUS) is required. 

## 5. TDP-43 Proteinopathy in the Fruit Fly

In 2006, TDP-43, which is encoded by the *TARDBP* gene, was identified as the major component of the ubiquitin-positive cytoplasmic inclusions found in ALS patients [31,253]. In 2008, mutations in *TARDBP* were linked to sporadic and familial ALS [254,255,256,257,258,259]. Currently, over 50 *TARDBP* mutations have been identified [260], which account for 4–5% of fALS and nearly 1% of sALS [261]. Importantly, in most if not all ALS patients, hyperphosphorylated, ubiquitinated, and truncated forms of TDP-43 are present in cytoplasmic insoluble aggregates, making TDP-43 proteinopathy a neuropathological hallmark of ALS [31]. 

TDP-43 is a member of the hnRNP family that is ubiquitously expressed. TDP-43, which acts as a transcriptional repressor, has been implicated in a wide variety of RNA metabolic processes including splicing, stability, and transport [262]. TDP-43 protein contains two RNA-recognition motifs (RRM1 and RRM2) and a C-terminal glycine-rich domain (GRD), as well as an NLS, an NES, and three mitochondrial localization motifs (Figure 3). In the physiological state, TDP-43 protein is mainly nuclear but shuttles between the nucleus and the cytoplasm, while, in ALS patients, TDP-43 exits the nucleus and forms cytoplasmic inclusions [31]. For this reason, it is thought that two nonexclusive mechanisms may account for the toxicity linked to TDP-43: an LOF due to the absence of TDP-43 in the nucleus and a GOF mechanism induced by TDP-43-containing aggregates [263]. TDP-43 is highly conserved during evolution [264], making *Drosophila* an ideal organism to decipher the function of TDP-43. The *Drosophila* ortholog of *TARDBP* is *TBPH*, and both LOF and GOF approaches have been modeled in *Drosophila* to gain insights into TDP-43 toxicity.

### 5.1. TBPH Is the Drosophila Ortholog of Human TARDBP

#### 5.1.1. *TBPH* Loss of Function

*TBPH*, which is expressed throughout development [265], was detected in neurons [266,267,268,269], glial cells [270,271], and muscles [265,266,270,272,273]. In neurons and glial cells, *TBPH* is mainly nuclear but was also found in the cytoplasm [266,270]. To better understand the role of *TBPH* in vivo, several groups have created *TBPH*-null mutant flies (Table 6). 

Depending on the *TBPH* mutant, lethality was observed at the second instar larval stage [268], at the pupal stage [275], or throughout development with few adult progeny eclosing [265,267,270,274]. One study reported that defective ecdysteroid receptor signaling may be the cause of late pupal death [276]. Altogether, these studies showed that *TBPH* is an essential gene. Adult flies lacking *TBPH* are morphologically normal [159,267], and they show a strongly reduced lifespan (3 to 10 days) and very poor climbing performance [159,266,267]. Larval locomotion is also affected by the absence of *TBPH*. The number of peristaltic waves [266,267] and the traveled distance [265,275,277,278] are severely reduced in mutant larvae compared to control. Larval locomotion impairment was not due to motoneuron loss, since their viability was not affected at this stage [279]. When *TBPH* was specifically expressed in motoneurons of *TBPH* mutant, larval locomotion was partially rescued [265,275,277]. Full rescue was obtained with a genomic fragment transgene carrying the *TBPH* gene [266]. Altogether, these data indicate that larval locomotion relies on the presence of *TBPH* not only in motoneurons but also in other tissues. However, no muscle alteration was detected in *TBPH* mutants [266,270,279].

Analyses at the larval NMJs of *TBPH* mutant have revealed contradictory results (Table 7).

Two groups found that the number of synaptic boutons and axonal branches was reduced [267,280], while another group found that both increased [265]. Four other studies did not detect any alteration in bouton and axonal branch numbers at the NMJs [159,266,277,281]. Thus, it is not clear whether larval locomotion impairment is due to morphology abnormalities at NMJs. Electrophysiological recordings performed by two groups revealed no difference in evoked excitatory junction potential (EJP) amplitude at *TBPH* mutant larval NMJs [266,278], while another group found a reduced amplitude of EJPs [282]. Nevertheless, both the amplitude and the frequency of miniEJPs were reduced, resulting in an increase of the quantal content [266,278]. These data strongly suggest that lack of *TBPH* may alter neurotransmission at NMJs, which may participate to the impaired larval locomotion. This idea is consistent with RNA-sequencing data showing that putative *TBPH* target genes are enriched for synaptic transmission, neurotransmitter secretion and endocytosis [275].

The presence of TDP-43 aggregates in glial cells of ALS patients [283,284,285] and the evidences that non-cell-autonomous mechanisms are at play in ALS disease [10] led to a better understanding of the role of *TBPH* in glial cells. In *TBPH* mutant, the number of glial cells does not seem to be affected in larval and adult central nervous system (CNS) [270,271]. However, peripheral glia failed to correctly wrap the motoneuron terminals at larval NMJs of *TBPH* mutants [271]. In addition, abnormal GluRIIA glutamate receptor clustering was observed at *TBPH* mutant larval NMJs [271]. Importantly, both mutant phenotypes were fully rescued by pan-glial expression of *TBPH* [271], confirming that *TBPH* is required to maintain glial integrity and glutamate receptor distribution. Interestingly, pan-glial expression of the glutamate transporter dEAAT1 in *TBPH* mutant restored the normal distribution of glutamate receptors but not the glial wrapping defects, suggesting that *TBPH* acts on different pathways in glial cells [271].

It was also described that *TBPH* mutants display an upregulation of retrotransposable elements in neural tissues [286]. By using an activity reporter of the small interfering RNA (siRNA) silencing machinery specifically expressed in motoneurons, the authors showed that *TBPH* mutants have a defective retrotransposon silencing. This was likely due to the reduced level of Dicer-2 observed in *TBPH* mutant as pan-neuronal expression of Dicer-2 prevented the upregulation of retrotransposable elements [286].

Another phenotype described in *TBPH* mutants was the decrease in dendritic branching of larval sensory neurons [274]. The same phenotype was observed in larvae expressing *TBPH* RNAi specifically in sensory neurons (Table 8), indicating that *TBPH* modulates dendritic branching cell-autonomously, as confirmed by mosaic analysis with a repressible cell marker (MARCM) analysis [274].

*TBPH* mutant adult escapers also have ectopic bristles and sensory neurons on their notum [287], indicating that the specification of sensory organ precursor (SOP) cells is altered in absence of *TBPH*. The precise generation of SOP cell number is regulated by microRNA (miR)-9a [294], which has reduced expression level in *TBPH* mutant [287]. Thus, it is likely that *TBPH* modulates miR-9a production to ensure proper neuronal specification. Another report has described that the wing phenotype induced by miR-1 overexpression was enhanced in heterozygous *TBPH* flies, suggesting that *TBPH* may dampen miR-1 activity [295].

Numerous studies have used RNA interference to examine the function of *TBPH* in different tissues (Table 8). While some divergences exist between the different experiments, certain conclusions may be drawn. First, the ubiquitous knockdown of *TPBH* was lethal with few adult escapers showing climbing defects [266,269,274], mimicking what was observed in *TBPH* mutant flies. Interestingly, when *TBPH* knockdown was induced at the adult stage, the flies had reduced lifespan and impaired climbing capacities, showing that *TBPH* is required throughout adult life [288]. Second, flies with pan-neuronal *TBPH* knockdown showed age-dependent impaired climbing and have reduced lifespan [266,267,269]. Similar phenotypes were described when *TBPH* expression was suppressed specifically in motoneurons [289]. Larval locomotion seems also affected by pan-neuronal or motoneuronal *TBPH* knockdown, depending on the RNAi and the Gal4 lines used [265,289]. Third, pan-muscle, pan-glial, or mushroom body *TBPH* downregulation gave opposite results, making it difficult to determine whether *TBPH* plays effectively an important role or not in such tissues [265,266,270,271,282,290]. Similarly, some studies reported that eye-specific *TBPH* knockdown has no effect or may induce a weak degeneration [269,272,290,292,293]. However, *TBPH* mutant adult escapers have a normal eye but survive only a few days, making it impossible to determine whether *TBPH* is clearly required for eye integrity [159,267].

#### 5.1.2. *TBPH* and TDP-43 Share the Same Functions

Several studies have shown that *TBPH* and TDP-43 share redundant functions and can substitute for each other. For example, rescue experiments of *TBPH* mutant phenotypes have shown that TDP-43 is as effective as *TBPH* for recovery of lifespan or locomotion defects [159,267,296]. To exclude any side effect due to overexpression, Chang and Morton used the clustered regularly interspaced short palindromic repeats (CRISPR)/Cas9 genome editing system to replace the *TBPH* gene by its human ortholog, allowing TDP-43 to be expressed at endogenous level. These flies had normal development and life span and they did not show any behavioral defects [297]. This clearly indicates that TDP-43 has the same function than *TBPH*, which is reinforced by overexpression studies (see below). Furthermore, one report showed that *TBPH* and TDP-43 share the ability to associate in vitro with Hrp38/Hrb98DE/CG9983 and Squid/Hrp40/CG16901 [298]. These two genes are the orthologs of human hnRNP A1 and A2/B1, which are known to interact with TDP-43 [299]. Thus, functional interaction of *TBPH*/TDP-43 with some hnRNPs is conserved during evolution. Moreover, TDP-43 and *TBPH* were shown to be interchangeable regarding their nucleic acid binding specificity and their role in splicing [300].

### 5.2. TBPH and TDP-43 Gain of Function Mutations Are Toxic

#### 5.2.1. Toxicity

*TBPH* and TDP-43 GOF experiments have demonstrated that a high level of *TBPH* or TDP-43 is very toxic (Table 9 and Table 10).

Pan-neuronal overexpression of TDP-43 or *TBPH* induced premature lethality [266,274,275,306,307], reduced lifespan [266,305,309,310], larval locomotion defect [266], and age-dependent climbing deficit [309,311]. Similar phenotypes were observed when *TBPH* or TDP-43 was expressed in motoneurons [265,273,275,289,290,293,302,305,306,313,315,316,317,318,319,320,322,323]. By using the temporal and regional gene expression targeting (TARGET) system, it was shown that adult-restricted overexpression of *TBPH* or TDP-43 in all neurons or motoneurons induced age progressive climbing defects and reduced lifespan [288,301,305,307,312,324]. This indicates that the level of TDP-43 must be tightly regulated throughout life. GOF experiments also revealed that excess of *TBPH* or TDP-43 is highly toxic in muscles [270,306,307], glial cells [270,307,309,325,326], and in the eye [266,274,289,290,292,293,302,303,304,306,307,310,312,314,315,316,317,318,319,320,324,327,328,329,330,331,332,333,334]. Moreover, it was reported that *TBPH* or TDP-43 overexpression induces cell death, as was shown in motoneurons [266,289,290,301], glial cells [309,326], mushroom bodies [265,290], and in the eye. At larval NMJs, TDP-43 or *TBPH* GOF gave some conflicting results (Table 11).

#### 5.2.2. *TBPH* and TDP-43 Gain-of-Function Phenotypes Are Dose- and Age-Dependent

It is important to keep in mind that all the phenotypes observed following *TBPH* or TDP-43 overexpression are dose-dependent, i.e., the severity of the phenotypes increases with higher levels of *TBPH*/TDP-43. For example, upon pan-neuronal expression, the reduction in lifespan is correlated with TDP-43 expression level, with a very high dose inducing lethality [305]. Similarly, eye degeneration is correlated with the expression levels of *TBPH*/TDP-43 that can lead to death at very high levels [289,293,315,333,336]. This may explain some discrepancies between different GOF studies (Table 9 and Table 10). Several phenotypes induced by *TBPH*/TDP-43 GOF, such as eye degeneration and climbing defects, show age-dependent progression [265,275,289,290,293,305,307,308,309,311,312,314,315,316,318,320,321,324,325,330,331,332,333,334]. As *TBPH* expression decreased with aging [338], one may speculate that reduced endogenous *TBPH* levels exacerbate GOF effects without excluding that phenotype aggravation may be due to continuous abnormal accumulation of *TBPH*/TDP-43.

#### 5.2.3. TDP-43/*TBPH* Toxicity Requires RNA Binding

An important point is that the toxicity of *TBPH*/TDP-43 is clearly associated with its RNA-binding property. For example, TDP-43 motoneuronal GOF causes reduced lifespan [293], age-dependent climbing deficit [305], and NMJ/axon degeneration of leg motoneurons [291]. These defects were not observed with TDP-43^RRM1mut^, a mutant form carrying mutations into the RRM1 domain, which abolish its RNA-binding capacity [291,293,305]. Similarly, TDP-43^RRM1mut^ GOF does not induce eye degeneration contrary to the wildtype form [293]. Importantly, the RNA-binding property of TPBH is also required to induce climbing defects upon pan-neuronal expression [298]. Altogether, these data show that RNA binding is crucial for *TBPH*/TDP-43 toxicity. One hypothesis is that *TBPH*/TDP-43 toxicity is linked to the nuclear functions of TDP-43 in RNA processing. However, RRM1 mutation abolished the strong toxicity of TDP-43 mutated in its NLS domain, which is mainly cytoplasmic [293]. Thus, it is very likely that TDP-43-associated toxicity requires RNA binding both in the nucleus and in the cytoplasm.

#### 5.2.4. TDP-43/*TBPH* Toxicity and Nucleocytoplasmic Localization

Overexpressed TDP-43^WT^ is predominantly present in the nucleus, although it was also detected in the cytoplasm of developing photoreceptors [289,307,336], motoneurons [296], and glial cells [336]. Interestingly, TDP-43 cytoplasmic localization extends until the larval NMJs when expressed in motoneurons [296] or glial cells [336], strongly suggesting that TDP-43 plays a local role at NMJs. Endogenous *TBPH*, which is mainly nuclear, was also found in the cytoplasm of neurons and glial cells [266,270]. Currently, no study has described the presence of *TBPH* at larval NMJs, maybe because its endogenous expression level is too low. Several groups have asked whether the subcellular localization of wildtype TDP-43 may influence its toxicity. To answer this issue, mutant forms of TDP-43 lacking the NLS or the NES were created (Table 12).

Accordingly, TDP-43^NLSmut^ and TDP-43^NESmut^ were predominantly cytoplasmic and nuclear, respectively [292,305,307,321]. Pan-neuronal overexpression experiments have shown that the wildtype TDP-43 form is more toxic than the cytoplasmic form, which is more toxic than the strict-nuclear form [305,307]. However, eye degeneration or age-dependent climbing defects, induced by eye or motoneuronal overexpression, were stronger with TDP-43^NLSmut^ compared to TDP-43^WT^, while TDP-43^NESmut^ had no effect [292,293,307,321,327]. In addition, one study found that TDP-43^WT^ or TDP-43^NLSmut^ GOF in muscles or glial cells was lethal but TDP-43^NESmut^ had no effect [307]. Altogether, these data indicate that cytoplasmic excess of TDP-43 is very toxic, and that nuclear accumulation of TDP-43 may be toxic depending on the cell type.

The presence of TDP-43-positive cytoplasmic inclusions in almost all ALS patients led to the hypothesis that they were directly linked to neurodegeneration. In *Drosophila*, cytoplasmic inclusions were not observed in neurons overexpressing TDP-43^NLSmut^, strongly suggesting that cytoplasmic inclusions are not absolutely required for toxicity [307]. From the studies cited above, it appears that accumulation of the nuclear restricted form of TDP-43 is less toxic than the WT or cytoplasmic forms. Fly neuronal expression of TDP-43^NESmut^, but not WT, was associated with TDP-43-positive nuclear inclusions, which very likely correspond to NBs [321]. NBs contain specific nuclear proteins and RNAs to modulate nuclear functions and homeostasis [339]. Interestingly, motoneuronal GOF-induced climbing defects associated with TDP-43 expression were worse with TDP-43^NLSmut^ compared to wildtype, and TDP-43^NESmut^ induced no defect [321]. Similar degrees of severity phenotypes were observed in the eye [321]. This suggests that TDP-43^NESmut^-associated NBs may be protective and may explain why nuclear accumulation of TDP-43 is less toxic.

#### 5.2.5. TDP-43 Toxicity of ALS-Linked TDP-43 Mutations

In order to better understand how TDP-43 mutations may induce motoneuronal death, several ALS-linked TDP-43 mutations have been studied in *Drosophila*. One study replaced the endogenous *TBPH* gene by *TARDBP* gene carrying either the G294A or M337V ALS-associated mutations [277]. Homozygous flies for each mutation had normal development and lifespan. In addition, the larval locomotion was unaltered, and their climbing capacity was only weakly reduced with aging compared to control flies and those expressing wildtype TDP-43 [277]. Interestingly, the two mutant proteins showed a higher level of cytoplasmic puncta in the adult brain compared to wildtype. Altogether, these data indicate that G294A and M337V mutations are not highly toxic, at least when expressed at endogenous levels [297]. In contrast, the overexpression of ALS-linked TDP-43 mutants (TDP-43^mutALS^) induces dramatic defects (Table 13).

Indeed, GOF of TDP-43^mutALS^ causes lethality, reduced lifespan, altered locomotion at larval and adult stages, and eye degeneration, depending on the tissue in which the mutant form was overexpressed. However, these defects were not so different from those observed with overexpression of wildtype TDP-43. In some cases, the defects were worse, and it was suggested that the ALS-linked mutations enhance the toxic function of TDP-43. In other cases, the mutant forms were a little less toxic and it was concluded that these ALS-linked mutations induce partial loss of TDP-43 function. Thus, it is still unclear how mutations of TDP-43 may induce motor deficit in the *Drosophila* ALS model.

However, a few reports found some differences between the wildtype and the mutated forms of TDP-43. One report described that the nuclei of motoneurons overexpressing mutants but not wildtype TDP-43 were misshapen, suggesting that nuclear stress may be associated with TDP-43 mutations [336]. When expressed in motoneurons, TDP-43^WT^ was detected in dynamic cytoplasmic granules present in axons and extending up to NMJs [296,336]. On the contrary, TDP-43^mutALS^ accumulated in the cell body and proximal axons due to impaired anterograde transport compared to wildtype [296]. In addition, the mobility of TDP-43^mutALS^ within cytoplasmic granules was highly reduced compared to wildtype [336]. Altogether, these data strongly suggest that TDP-43^mutALS^ are not transported correctly along axons and that the delivery of TDP-43-interacting mRNAs to distal neurites may be compromised by ALS-linked mutations into TDP-43.

### 5.3. Molecular Mechanisms Underlying TDP-43 Toxicity

#### 5.3.1. Splicing Repression

RNA-sequencing of *TBPH* mutant heads showed cryptic exon incorporation, suggesting impaired splicing repression [280]. To go further, the authors used a chimeric construct consisting of the N-terminal region of TDP-43, including the two RNA-binding domains, fused to the splicing repressor ribonucleoprotein, PTB-binding 1 (RAVER1) (TDP-43^Nter^-RAVER1). When TDP-43^Nter^-RAVER1 was expressed in motoneurons of *TBPH* mutants, it highly extended lifespan [280]. On the contrary, the TDP-43^Nter^-RAVER1 construct carrying two mutations in the RRM1 that abolish RNA binding had no effect [280]. This confirms that the protective effect of TDP-43^Nter^-RAVER1 was due to TDP-43 binding onto its target genes. Altogether, these data strongly suggest that a major function of *TBPH* is splicing repression, which is very likely involved in the drastic phenotypes induced by loss of *TBPH*.

#### 5.3.2. Mitochondrial Dysfunction

Abnormal fragmentation of mitochondria was described in the adult brain upon pan-neuronal expression of TDP-43 [311]. Highly fragmented mitochondria were also observed in thoracic muscles and axons of leg motoneurons expressing TDP-43 [185]. Fusion of mitochondria is promoted by Mitofusin/Marf, which has reduced expression level in fly heads overexpressing TDP-43 [185,311]. This suggests that TDP-43 GOF alters mitochondrial dynamic by inducing excessive fission of mitochondria due to Mitofusin/Marf downregulation. When overexpressed in the eye, TDP-43^WT^ or TDP-43^A315T^ induced mitochondrial damages including reduced size and abnormal cristae [312]. In addition, abnormally elevated ROS levels were detected in larval motoneurons overexpressing TDP-43^WT^ or TDP-43^A315T^ [312]. Pan-neuronal TDP-43^WT^ or TDP-43^A315T^ GOF induced an activation of the mitochondrial unfolded protein response (mitoUPR), characterized by the increased expression of known genes involved in mitoUPR, including *Lon*, the *Drosophila* ortholog of *LonP1* [312]. LonP1 is a mitochondrial protease that plays an important role in mitochondrial protein quality control. *Lon* silencing exacerbated TDP-43-induced retinal degeneration and mitochondrial damages, as well as increased the level of mitochondrial TDP-43 without affecting the cytoplasmic level [312]. This suggests that Lon plays a protective role against TDP-43 proteinopathy. Altogether, these data strongly suggest that mitochondrial dysfunction may be associated with TDP-43 excess and that reversing mitochondrial damages may provide a potential therapeutic approach.

#### 5.3.3. Transposon Upregulation

RNA-sequencing experiments have shown that pan-neuronal or pan-glial expression of TDP-43 induced upregulation of some retrotransposable element (RTE) in adult brain [309]. Then, the authors focused on *gypsy* RTE, for which level was highly increased in response to TDP-43 expression in glial cells. This was due to a reduction in the siRNA pathway, which is the major post-transcriptional silencing component of RTE in somatic tissue. Interestingly, reducing the glial expression of *gypsy* by RNA interference ameliorated the lifespan alteration induced by TDP-43 glial expression, strongly suggesting that loss of *gypsy* silencing was at least partially involved in TDP-43-induced toxicity [309]. This was further confirmed by the finding that glial cells undergo programmed cell death due to DNA damage caused by TDP-43-induced RTE replication [309]. In addition, DNA damage and apoptosis of nearby neurons were also observed upon TDP-43 glial GOF, suggesting that glial toxicity may spread to adjacent neurons [326]. Altogether, these studies suggest that TDP-43 overexpression alters the silencing of RTE, leading to DNA damage and subsequently cell death, which may contribute to neurodegeneration processes in ALS. Interestingly, upregulation of RTE expression was also reported in *TBPH* mutant [286], showing that TDP-43 GOF and *TBPH* LOF share similar phenotypes.

#### 5.3.4. Excitotoxicity

Two-dimensional gel electrophoresis followed by mass spectrometry analyses on *TBPH* mutant heads showed that the levels of glutamic acid decarboxylase (Gad1) protein were reduced [342]. This downregulation occurred also at the mRNA level. Pan-neuronal expression of Gad1 in *TBPH* mutant partially restored the defects in larval locomotion and the abnormal distribution of the post-synaptic GluRIIA glutamate receptors and Discs-large (Dlg) scaffolding protein. On the contrary, pan-neuronal silencing of *Gad1* triggered larval locomotion defects and abnormal organization of GluRIIA and Dlg, as was observed in *TBPH* mutants. Interestingly, pan-glial expression of *Gad1* completely rescued larval locomotion defects and abnormal GluRIIA distribution of *TBPH* mutants but not the Dlg one. Conversely, *Gad1* silencing in glial cells induced locomotion defects and abnormal GluRIIA distribution but did not affect Dlg. Increased glutamate levels were found in the hemolymph of *TBPH* mutant or *Gad1* knockdown larvae [342], suggesting that an abnormal concentration of the neurotransmitter glutamate may be responsible for these defects. Thus, it would be conceivable to pharmacologically modulate glutamate signaling to interfere with neurodegenerative progression in ALS patients.

### 5.4. Genetic Modifiers of TDP-43 Toxicity

#### 5.4.1. Stress Granules

Kim et al. found that *Drosophila* pan-neuronal TDP-43 expression resulted in increased phosphorylation of eIF2α [314]. In response to cellular stress, several kinases phosphorylate eIF2α, leading to translation repression and SG formation [343,344]. It was also shown that TDP-43 is recruited to SG upon stress [153], suggesting that TDP-43 may confer toxicity by increasing SG accumulation. RNAi-induced knockdown or pharmacological inhibition of poly(ADP-ribose)ylation glycohydrolase (PEK), a kinase that phosphorylates eIF2α, suppressed the climbing deficit induced by TDP-43. Conversely, downregulation of Gadd34, a phosphatase of eIF2α, enhanced the locomotor defects [314]. Altogether, these data showed that modulating the phosphorylation level of eIF2α might be therapeutically interesting to mitigate TDP-43 toxicity.

Using a candidate approach to search for RBPs that modulate TDP-43 toxicity, Coyne et al. identified *dFMRP* [318], the *Drosophila* ortholog of *FMRP*, which encodes a translational regulator and component of neuronal RNA granules [345]. LOF and GOF of *dFMRP* enhanced and reduced TDP-43-induced eye degeneration and larval locomotor dysfunction, respectively [318]. In primary larval motoneuron cultures, TDP-43 was detected in some cytoplasmic puncta, which were positive for dFMRP and other markers of SGs. In addition, immunoprecipitation experiments from adult heads indicated that TDP-43 associates to endogenous dFMRP in vivo. Fractionation experiments showed that *dFMRP* GOF decreased the insoluble fraction of TDP-43, suggesting that excess of dFMRP may modify TDP-43 aggregation properties to restore translation [318]. Thus, this study indicates that dFMRP may mitigate TDP-43 toxicity by remodeling RNA granules and restoring translation.

Ataxin-2 is known to participate to SG assembly [346,347]. In *Drosophila*, *dAtx2* GOF aggravated the lifespan defects and eye degeneration induced by TDP-43. On the contrary, reducing *dAtx2* dose rescued these phenotypes [308]. From these observations, one may conclude that excess of Atx-2 may exacerbate TDP-43 toxicity. Accordingly, reduction of the Atx-2 level has beneficial effects in a mouse model of TDP-43 proteinopathy [348].

The poly(A)-binding protein nuclear 1 (PABPN1) was identified as a partner of TDP-43 [331]. PABPN1 (also known as PABP2) is a ubiquitous protein that controls the poly(A) tail length of RNA transcripts [349]. Furthermore, PABPN1 was shown to form a complex with TDP-43 and FUS in mammalian cells [350]. LOF of *PABP2*, the *Drosophila* ortholog of *PABPN1*, enhanced the eye degeneration and the larval locomotor defect induced by TDP-43 GOF [331]. In primary motoneuron cultures, TDP-43 disturbed SG dynamics, and this defect was suppressed by PABPN1 co-expression [331]. Thus, PABP2 might modify TDP-43 toxicity by modulating SG dynamics. Another study found that loss of *PABP2* slightly increased the median lifespan of flies expressing TDP-43 in motoneurons [313], suggesting a different role during aging.

PolyADP ribosylation (PARylation) is a reversible PTM in which polymers of ADP-ribose are attached to proteins by PAR polymerase enzymes (PARPs) and removed by PAR glycohydrolases (PARG). PARylation was shown to regulate SG dynamics with a decreased level suppressing SG formation [351]. In *Drosophila*, both *Parp* knockdown and *Parg* overexpression suppressed the eye degeneration caused by TDP-43 GOF [351]. *Parp* silencing also rescued age progressive climbing defects and reduced lifespan of pan-neuronal TDP-43 expressing flies [351]. A genetic screen based on the modification of the rough eye phenotype induced by TDP-43 GOF showed that downregulation of *tankyrase* (*Tnks*), which encodes a PARP, restored eye structure [310]. Moreover, *Tnks* knockdown rescued the lifespan defect induced upon pan-neuronal TDP-43 expression. Interestingly, the authors found PAR-binding motifs in the NLS region of TDP-43 that are required to localize TDP-43 to SGs [310]. They also found that *Tnks* downregulation induced a redistribution of TDP-43 protein, with an increase in the nucleus and a decrease in the cytoplasm. This was likely due to an impairment of TDP-43 incorporation into SGs. Altogether, these studies showed that decreased PARylation is protective against TDP-43 toxicity.

The discovery that modulation of SG dynamics may modify the TDP-43-induced phenotypes opens the way to future studies targeting SGs for potential therapeutic strategy.

#### 5.4.2. Cytoplasmic Aggregation

Choksi et al. found that TDP-43^Q331K^ mutant, but not wildtype or M337V mutant, was detected in cytoplasmic puncta when expressed in the developing eye [306]. Co-expression of TDP-43^Q331K^ with *doubletime*, the fly ortholog of casein kinase Iε, strongly enhanced the degenerative eye phenotype induced by TDP-43^Q331K^ but not the one induced by wildtype or M337V TDP-43 [306]. Interestingly, when co-expressed with *doubletime*, the Q331K mutant showed a higher level of S409/410 phosphorylation, which is detected in cytoplasmic inclusions of ALS patients [352]. In addition, high-molecular-weight oligomeric species were detected upon Q331K expression, and they were enhanced when *doubletime* was co-expressed [306]. These data suggest that the toxicity of TDP-43^Q331K^ was linked to abnormal phosphorylation and cytoplasmic aggregation. However, the reason why Q331K was more toxic compared to wildtype or M337V is still unclear. Nevertheless, this study suggests that targeting a specific kinase to modulate TDP-43 phosphorylation may be therapeutically interesting.

Budini et al. developed a cellular model of TDP-43 aggregation by using a GFP-tagged construct composed of repetitions of the Q/N-rich C-terminal region of TDP-43 (GFP-12xQ/N) [353]. In cell lines, the co-expression of TDP-43 and GFP-12xQ/N triggered the formation of TDP-43 aggregates, which were cytoplasmic, ubiquitinated, and phosphorylated, as was observed in ALS patients [31,253]. *TBPH* overexpression induced strong neurodegeneration in the eye that was completely abolished when GFP-12xQ/N was co-expressed [304]. In addition, *TBPH* and GFP-12xQ/N were found co-localized in cytoplasmic aggregates in retinal cells. Biochemical fractionation experiments indicated that *TBPH* was mainly present in the soluble fraction upon eye expression, while it was predominantly present in the insoluble fraction when co-expressed with GFP-12xQ/N [304]. Altogether, these data strongly suggest that the excess of soluble *TBPH* is toxic and that aggregates have a protective effect, likely by sequestrating excess of *TBPH*. The same group showed that pan-neuronal expression of GFP-12xQ/N induced locomotion defects in mid-adult life [338]. They also found that endogenous *TBPH* expression dropped during aging, which likely explains why *TBPH* sequestration by GFP-12xQ/N was deleterious during aging [338].

By using a proximity-dependent biotin identification approach, Chou et al. found that components of the nuclear pore complex and nucleocytoplasmic transport machinery were enriched in detergent-insoluble TDP-43 aggregates [317]. They also showed that TDP-43 aggregates sequestered and/or mislocalized nucleoporins (Nup). To test for functional interaction, the authors asked whether Nup function might modify TDP-43 toxicity in flies. They found that LOF of *Nup50, Nup93*, *Nup98-96*, *Nup107*, or *Nup214* suppressed the TDP-43-mediated rough eye phenotype and rescued the larval locomotor phenotype induced by motoneuronal TDP-43 GOF [317]. This study suggests that some nucleocytoplasmic transport alteration may be part of TDP-43 toxicity. Another group found that mutation in *Nup50* rescued lifespan defects of motoneuronal TDP-43-expressing flies [313]. Interestingly, TDP-43 was still predominantly nuclear in larval neurons lacking *Nup50* [313], suggesting that the protective effect of *Nup50* mutation was not due to a major nucleocytoplasmic redistribution of TDP-43 but rather to nucleocytoplasmic transport modulations. Thus, targeting some Nup proteins may have a beneficial effect on ALS disease.

#### 5.4.3. Unfolded Protein Response

The co-expression of TDP-43 with a GFP reporter of the unfolded protein response revealed that ER stress was elevated in *Drosophila* retinal cells compared to control [324]. Then, the authors asked whether Clusterin, a normally secreted chaperone that is redirected to the cytosol under ER stress condition [354], might modulate TDP-43-induced toxicity. Note that Clusterin has no *Drosophila* ortholog. When expressed in adult fly motoneurons, TDP-43 was found in cytoplasmic foci distributed in soma and neuronal processes. In contrast, co-expression of Clusterin restored the predominant nuclear localization of TDP-43 [324]. In addition, co-expression of Clusterin with TDP-43 partially restored the climbing defect, the reduced lifespan, and the eye degeneration induced by TDP-43 GOF [324]. Thus, this study suggests that Clusterin may be protective upon TDP-43-induced ER stress.

The deubiquitinating enzyme UBPY (ubiquitin isopeptidase Y) was identified as an interactor of TDP-43 [332]. The knockdown of its *Drosophila* ortholog, *dUBPY* (also known as *Ubiquitin specific protease 8*) resulted in higher amounts of insoluble and likely ubiquitinated TDP-43 in fly heads [332]. Additionally, *dUBPY* silencing enhanced the age-dependent eye degeneration induced by TDP-43 expression [332]. Another study found that the TDP-43^M337V^-induced eye degeneration was suppressed or enhanced by GOF and LOF of CG5445 gene, respectively [334]. CG5445 encodes a protein that interacts with ubiquitinated proteins and promotes their degradation [334]. Accordingly, the authors found that CG5445 GOF and LOF increased or decreased, respectively, the soluble form of TDP-43^M337V^, likely facilitating its degradation.

Altogether, these findings showed that modulating the unfolded protein response and ubiquitination might influence TDP-43 aggregation and toxicity, which may open the way for the discovery of potential therapeutic strategy.

#### 5.4.4. Inflammation

A genetic screening, aiming to find modifiers of reduced lifespan induced by motoneuronal expression of TDP-43, identified the mitogen-activated protein kinase kinase kinase (MAP3K) Wallenda (Wnd), the *Drosophila* ortholog of dual leucine kinase (DLK) [323]. *Wnd* LOF and GOF extended and reduced lifespan, respectively. Effector kinases downstream of Wnd are p38 and JNK, two stress-activated kinases. The JNK pathway had a protective effect as the GOF of *Basket*, the *JNK Drosophila* ortholog, extended lifespan of flies expressing TDP-43 in motoneurons. Conversely, a reduced dose of *Basket* reduced lifespan. Contrary results were obtained with *p38*b, one the three *p38 Drosophila* orthologs, indicating that the p38 pathway was rather deleterious. This report also showed that oxidative stress and neuroinflammation were differentially modulated by *Basket* and *p38b* [323], suggesting that these two processes may be part of TDP-43 toxicity.

*Drosophila* pan-glial TDP-43 increased the expression of the inflammatory genes *Dorsal* (the ortholog of human NF-kappa B,NF-κB and inducible Nitric Oxyde Synthase, iNOS) [325]. This was rescued by RNAi-induced glial knockdown of *Ptp61F*, the *Drosophila* ortholog of human *Ptp1b* encoding tyrosine phosphatase 1B, which is a ubiquitous enzyme anchored in the ER membrane, upregulated in neuroinflammatory conditions [355]. In addition, *Ptp61F* silencing mitigated the age-dependent climbing defects and the reduced lifespan induced by glial GOF of TDP-43 [325]. This study suggests that the *Ptp1b*-dependent inflammatory response may be associated with TDP-43 toxicity.

Thus, inflammation processes may represent a target to counteract disease progression in ALS.

#### 5.4.5. Mammalian Target of Rapamycin (mTOR) Pathway

In *TBPH* mutant larvae, the phosphorylation levels of ribosomal protein S6 kinase (S6K) and eukaryotic translation initiation factor 4E (4E-BP1), two downstream effectors of mTORC1 [356], were decreased [174]. In addition, the expression level of *raptor* mRNA was decreased in *TPBH* mutants. Raptor is an essential component of mTORC1 that regulates mTORC1 lysosomal localization. *TBPH* mutants also displayed an upregulation of some lysosomal and autophagic genes, as well as alteration in autophagosome–lysosome fusion [174]. Administration of the mTOR inhibitor rapamycin to *TBPH* mutant aggravated larval locomotion. On the contrary, the mTOR agonist phosphatic acid ameliorated this phenotype. In both cases, the effects of rapamycin or phosphatic acid were dose-dependent [174]. Another report found that rapamycin administration ameliorated the reduced lifespan and locomotion defects induced by adult motoneuronal *TBPH* GOF [301]. Altogether, these studies suggest that impaired mTORC1 signaling influences TDP-43 toxicity and that restoring mTOR signaling may be beneficial.

#### 5.4.6. Autophagosomes

A genome-wide RNAi screening identified the inositol-1,4,5-trisphosphate receptor type 1 (ITPR1) as a strong modulator of TDP-43 subcellular localization [322]. The silencing of ITPR1, which is an inositol 1,4,5-trisphosphate (IP3)-gated Ca^2+^ channel localized in the endoplasmic reticulum, promoted cytoplasmic accumulation of TDP-43 and potentiated its recruitment to phagosomes [322]. This suggests that ITPR1 silencing promotes TDP-43 clearance by autophagosomes. Mutations in *Itp-r83A*, the only fly ortholog of *ITPR1*, increased the cytosolic fraction of TDP-43 in larval neurons and rescued the climbing defects and the reduced lifespan induced by motoneuronal expression of TDP-43 [322]. Thus, it is likely that phagosome metabolism can influence TDP-43 toxicity, and future studies will be needed to determine whether modulating phagosome function may have a therapeutic effect.

#### 5.4.7. Chaperone

Co-expression of HSP67Bc, the *Drosophila* ortholog of human *HSPB8*, rescued the strong eye degenerative phenotype induced by TDP-43 mutated in its NLS domain (TDP-43^NLSmut^) but had no effect on the rough eye phenotype induced by wildtype TDP-43 [327]. HSP67Bc facilitates the degradation of misfolded proteins, which likely explains its protective effect as the protein level of TDP-43^NLSmut^ but not wildtype was reduced when they were co-expressed. Conversely, RNAi-induced knockdown of *HSP67Bc* aggravated the TDP-43^NLSmut^-associated eye phenotype, which was correlated with a high accumulation of ubiquitinated proteins [327]. Thus, HSP67Bc is protective against the abnormal protein homeostasis induced by TDP-43^NLSmut^.

#### 5.4.8. Chromatin

Berson et al. selected some genes related to various aspects of chromatin regulation and asked whether their downregulation may modify the TDP-43-induced rough eye phenotype [330]. They found that TDP-43-induced toxicity was strongly enhanced by knockdown of *Chd1*, which encodes a nucleosome-remodeling enzyme required for heat-shock gene expression following stress [357]. Upon heat stress, the upregulation of several heat-shock genes was lower in flies expressing TDP-43, and this defect was abolished by *Chd1* co-expression [330]. Co-immunoprecipitation experiments on whole-fly lysates indicated that TDP-43 interacts with Chd1. In addition, chromatin immunoprecipitation (Chip)-qPCR analyses showed that TDP-43 impaired the recruitment of Chd1 to chromatin [330]. These data suggest that TDP-43 may prevent the proper recruitment of Chd1 to chromatin, leading to dampened stress-related gene expression. Thus, modulating chromatin dynamics may be an interesting way to fight against cellular stress induced by TDP-43.

#### 5.4.9. hnRNPs

Appocher et al. investigated whether RNAi-induced knockdown of *Drosophila* hnRNPs may modify the degenerative phenotype induced by *TBPH* expression in the eye. In this way, Hrb27c, CG42458, Glo, Syncrip/Syp, and Hrp38 were identified as strong suppressors, while Hrb87F, Sm, Heph, and Rump were mild suppressors of eye degeneration [303]. Thus, *TBPH* toxicity may be modulated by hnRNPs.

#### 5.4.10. Metabolic Deregulation

Clinical observations indicate that ALS patients have metabolic disturbances including body weight loss [358], hypermetabolism [359,360], glucose intolerance [361], and defects in lipid metabolism [362]. Metabolomic analyses performed on whole larvae revealed that glucose metabolism was increased when TDP-43 (wildtype or G298S) was expressed in motoneurons [363]. In addition, mRNA levels of phosphofructokinase, an enzyme that control the rate of glycolysis, was increased, strongly suggesting that glycolysis was upregulated by TDP-43 motoneuronal expression. By using a glucose sensor, it was found that the entry of glucose in motoneurons was enhanced by the expression of TDP-43 G298S but not wildtype [363]. Interestingly, a high glucose diet was sufficient to mitigate the larval locomotor defect, as well as the reduced survival induced by TDP-43 (wildtype or G298S) expression, in motoneurons. In addition, motoneuronal co-expression of the human glucose transporter GLUT-3 suppressed the larval locomotor defects, as well as the alterations at larval NMJs induced by TDP-43 [363]. These data strongly suggest that increasing glucose availability is protective against the deleterious effect of TDP-43.

Some alterations of lipid metabolism, such as a decrease in the carnitine shuttle and reduced lipid beta oxidation, were also found in larvae expressing TDP-43 (wildtype or G298S) in motoneurons [364]. In addition, the mRNA levels of some carnitine shuttle components were misregulated by TDP-43. To bypass the carnitine shuttle, medium-chain fatty acids or beta-hydroxybutyrate were administrated in the food, and this was sufficient to mitigate the larval locomotor defects induced by TDP-43 [364].

Altogether, these studies showed that dietary intervention might be an interesting way to alleviate TDP-43 induced defects.

### 5.5. Target RNAs

One challenge when studying RBPs is to identify their target RNAs. Several RNA targets of *TBPH* have been identified in *Drosophila* (see also [276,365,366]).

#### 5.5.1. Futsch

One report found that the level of Futsch protein, a neuronal microtubule-binding protein homolog to human Microtubule associated protein 1B (MAP1B), was decreased in *TBPH* mutant heads and at larval NMJs [279]. Immunoprecipitation from heads expressing pan-neuronally Flag-tagged TPBH showed that *futsch* mRNA was highly enriched [279]. In addition, an RNA-binding defective *TBPH* construct was not able to restore the endogenous Futsch level as was observed with wildtype, indicating that the RNA-binding activity of *TBPH* was required to maintain *futsch* expression level [279]. Altogether, these data strongly suggest that *TBPH* binds the *futsch* transcript to positively regulate its expression. The *futsch* mRNA was also enriched in immunoprecipitation experiments on flies expressing YFP-tagged TDP-43 in the eye [335], indicating that both *TBPH* and TDP-43 may interact with *futsch* mRNA. When TDP-43 was expressed in motoneurons, the levels of *futsch* mRNA and protein were increased in the cell bodies, while they were decreased at the NMJs compared to control [335], suggesting a failure in *futsch* mRNA transport. In addition, polysome fractionation experiments indicated that TDP-43 induces the *futsch* mRNA to shift toward the untranslated fractions, strongly suggesting that TDP-43 represses the translation of *fustch* mRNA [335]. This is in agreement with RNA-sequencing data that showed a downregulation of *futsch* in *TBPH* GOF and no change in LOF condition [275]. It was shown that both *TBPH* and TDP-43 directly bind the 5′-UTR of *futsch* mRNA [367], which contains UG-rich sequences known to be target of TDP-43. A luciferase assay performed with the 5′-UTR of the *futsch* mRNA region suggested that *TBPH* positively modulates the translational efficiency of *futsch* [367]. It is noteworthy that no evidence for a repressor role of TDP-43 was provided in this study.

#### 5.5.2. Histone Deacetylase 6 (HDAC6)

HDAC6 is a cytoplasmic deacetylase that plays an important role in the detection and degradation of ubiquitinated cellular aggregates [368]. In *TBPH* mutants, *HDAC6* mRNA level was strongly decreased [268,340], while TDP-43 motoneuronal GOF increased *HDAC6* mRNA level [340]. In addition, TDP-43 and *TBPH* bound *HDAC6* mRNA [268,279]. Interestingly, HDAC6 is necessary and sufficient for deacetylation of Bruchpilot (Brp) [340], an important player at presynaptic density that tethers vesicles [369]. Thus, it is tempting to speculate that HDAC6 dysregulation observed upon TDP-43 GOF or in *TBPH* LOF might alter Brp acetylation and synaptic transmission.

#### 5.5.3. Cacophony

Transcriptome analysis revealed that the splicing of *cacophony* transcripts was altered in *TBPH* mutant CNS [275]. The *cacophony* gene codes for a voltage-gated calcium channel localized at the active zone of NMJs that is required for neurotransmitter release. The level of cacophony protein was reduced in *TBPH* mutant larval NMJs, which have abnormal crawling behavior [277]. Interestingly, pan-neuronal or motoneuronal expression of *cacophony* fully rescues the locomotion behavior phenotype but not the pupal lethality associated with *TBPH* mutation, suggesting that the locomotion phenotype is mainly due to the lack of cacophony protein in motoneurons [277]. However, another study reported that *cacophony* may also be required in a few central neurons [278]. RT-PCR experiments on immunoprecipitates from adult fly using an anti-*TBPH* antibody revealed the presence of *cacophony* transcripts, strongly suggesting that *TPBH* binds *cacophony* mRNA [277]. In addition, Lembke et al. showed that the subtle changes in the splicing pattern of *cacophony* observed in *TBPH* mutant decreased the cacophony protein expression level [370]. Altogether, these studies strongly suggested that *TBPH* is required for proper splicing of *cacophony* transcripts. Thus, these findings revealed that the splicing alteration of genes involved in synaptic transmission is a consequence of *TBPH* dysfunction, which might open new ways to better understand ALS pathogenesis.

## 6. Conclusions

*Drosophila* is a powerful genetic tool that has made major contributions to further our understanding of several neurodegenerative disorders, including ALS (Figure 2). Furthermore, large-scale screenings in *Drosophila* have allowed the identification of numerous ALS disease modifiers, paving the way for future studies in mammals. Genome-wide association studies and whole-exome/genome sequencing have accelerated the pace of identification of new ALS-associated genes, as illustrated for instance by the recent discovery of ALS-linked mutations in the *kinesin family member 5A* gene [371,372]. Moreover, large-scale analysis using next-generation sequencing has significantly increased the rate of new mutation detection in already known ALS-causing genes. The subsequent challenging question relates to the pathogenicity of these new variants. The wide variety of genetic tools available in *Drosophila* combined with their short lifespan will undoubtedly help to gain insights into the pathogenic mechanisms associated with these novel genes or variants.

Despite the well-recognized motoneuronal death in ALS, motoneuron neighboring cells including astrocytes, oligodendrocytes, and interneurons degenerate before motoneuron loss [10], strongly suggesting that they contribute to ALS pathogenesis. Recently, one report showed that TDP-43 overexpression in glial cells induced neuronal death in adult brains [326], showing that non-cell-autonomous mechanisms also take place in flies. However, this non-cell-autonomous degeneration of motoneurons is still a neglected subject of research in flies, despite all the genetic tools that are available in *Drosophila*.

Despite intensive research over the past decades, no efficient therapy is currently available for ALS. *Drosophila* will certainly help to identify and characterize novel molecular and cellular mechanisms involved in this disease, giving hope for the development of therapeutic strategies.

## Figures and Tables

**Table 1:** Phenotypes observed in *dSod1* (*superoxide dismutase-1)* mutant backgrounds.

Mutant Line	Phenotype	Reference
*dSod1^n108^*	Homozygous lethality with rare eclosing adults, sterile, and early dying within 2–3 days No detectable superoxide dismutase activity Hypersensitivity to paraquat	[49]
	Necrotic lesions throughout retina	[50]
*dSod1^x16^*	No detectable superoxide dismutase activity	[50]
	Partially lethal	[51]
*dSod1^x139^*	No detectable superoxide dismutase activity	[50]
	Partially lethal	[51]
	Reduced eclosion rate, eclosing adults with shorter lifespan	[54,55]
*dSod1^x16^/dSod1^x139^*	Very reduced lifespan Impaired adult locomotion	[56]
*dSod1^G37R^*	No lethality, adult eclosion as wildtype, no adult locomotion defect.	[57]
*dSod1^G51S^*	Impaired larval crawling Reduced adult viability, adult escapers with locomotor defect	[57]
*dSod1^G85R^*	Impaired larval crawling Reduced adult viability, mostly lethal at pharate stage Adult escapers with muscle atrophy and denervation	[57]
	Reduction in NMJ bouton number and electrophysiological defect	[58]
*dSod1^H48R^*	Reduced adult viability, mostly lethal at pharate stage	[57]
*dSod1^H71Y^*	Impaired larval crawling Reduced adult viability, mostly lethal at pharate stage, adult escapers with locomotor defect, muscle atrophy, and denervation	[57]

This table describes the phenotypes associated with different *dSod1* mutant lines. Note that the experiments could be done at different temperatures.

**Table 2:** Gain-of-function phenotypes induced by hSOD1. WT, wildtype.

Gal4 Line	UAS Line	Phenotype/Reference
D42-Gal4 (motoneurons)	hSOD1 WT	No effect on lifespan, progressive motor dysfunction [74,75] Abnormal synaptic transmission [74]
	hSOD1 A4V	No effect on lifespan, progressive motor dysfunction [74]
	hSOD1 G85R	No effect on lifespan, progressive motor dysfunction [74,75] Abnormal synaptic transmission [74] Induction of stress response in glial cells [74]
D42-Gal4 (motoneurons) 3 mM BMAA (β-*N*-methylamino-l-alanine)	hSOD1 WT	Increased lifespan compared to control flies [76]
	hSOD1 A4V	Increased lifespan compared to control flies [76]
	hSOD1 G85R	Increased lifespan compared to control flies [76]
M1B-Gal4 (glial cells) 3 mM BMAA (β-*N*-methylamino-l-alanine)	hSOD1 WT	Accelerated death compared to control flies [76]
	hSOD1 A4V	Accelerated death compared to control flies [76]
	hSOD1 G85R	Accelerated death compared to control flies [76]
24B-Gal4 (muscles)	hSOD1 WT	No phenotype in thoracic muscle fibers, Slight motor behavior defect and normal lifespan [77]
	hSOD1 G93A	Upheld wings, swollen mitochondria, Impaired motor behavior and reduced lifespan [77]

This table describes the phenotypes associated with different UAS lines. Note that the experiments could be done at different temperatures.

**Table 3:** Gain-of-function phenotypes induced by FUS. CNS, central nervous system.

Gal4 Line	UAS Line	Phenotype/Reference
Act5C-Gal4 (ubiquitous)	FUS WT	Lethal, no eclosion [160]
	FUS R521G	Lethal, no eclosion [160,161]
	FUS R521H	Lethal, no offspring [161]
	FUS P525L	Lethal, no offspring [161]
	FUS Δ32	No effect on viability [160]
Tubulin-Gal4 (ubiquitous)	FUS WT	Lethal, no offspring [161]
	FUS R521G	Lethal, no offspring [161]
	FUS R521H	Lethal, no offspring [161]
	FUS P525L	Lethal, no offspring [161]
Tubulin-Gal4 Tubulin-Gal80TS (expression induced at adult stage)	FUS WT	Severe reduction of lifespan [161]
	FUS R521G	Severe reduction of lifespan [161]
	FUS R521H	Severe reduction of lifespan [161]
	FUS P525L	Severe reduction of lifespan [161]
Appl-Gal4 (pan-neuronal)	FUS WT	Normal eclosion [158]
	FUS R518K	Pupal lethality [158]
	FUS R521C	Pupal lethality [158]
	FUS R521H	Pupal lethality [158]
Elav-Gal4 (pan-neuronal)	FUS WT	Rescued eclosion and locomotion in *caz^1^* mutants [159] Lethal pupal [162] Reduced viability at 25 °C, improved at 19 °C [160] Lethal, no offspring [161]
	FUS R521G	Reduced viability at 25 °C, improved at 19 °C [160] Lethal, no offspring [161]
	FUS R521H	Lethal, no offspring [161]
	FUS P525L	Rescued eclosion in *caz^1^* mutants [159] Lethal, no offspring [161]
	FUSΔ32	No effect on viability [160]
ElavGS (pan-neuronal, inducible)	FUS WT	RU486 treatment at eclosion, decline in lifespan, 50% lethality at 28 days [158] Decreased lifespan, no degeneration in brain [162]
	FUS R521C	RU486 treatment at eclosion, 50% lethality at 10 days, impairment in climbing [158]
D42-Gal4 (motoneuron)	FUS WT	Reduced viability at 25 °C, improved at 19 °C, defect in adult climbing [160] Mitochondria defect [163] Defect in adult eclosion [164] Eclosion defect and escapers with immature phenotype and reduced lifespan [161]
	FUS R495X	No effect on adult eclosion [164]
	FUS R521G	Reduced viability at 25 °C, improved at 19 °C, defect in adult climbing [160] Eclosion defect and escapers with immature phenotype and reduced lifespan [161]
	FUS R521H	Eclosion defect and escapers with immature phenotype and reduced lifespan [161]
	FUS P525L	Mitochondria defect [163] Eclosion defect and escapers with immature phenotype and reduced lifespan [161]
	FUS Δ32	No effect on viability and adult climbing [160]
D42-Gal4 Tubulin-Gal80TS (expression induced at adult stage)	FUS WT	Reduced lifespan and impaired flight ability [161]
	FUS R521G	Reduced lifespan and impaired flight ability [161]
	FUS R521H	Reduced lifespan and impaired flight ability [161]
	FUS P525L	Reduced lifespan and impaired flight ability [161]
OK371-Gal4 (glutamatergic neurons)	FUS WT	Mild larval crawling defect, no effect on synaptic boutons [158] Disruption in motoneuron cluster, decreased synaptic bouton number, reduction in mobility [147] Larval locomotion activity impaired, decreased synaptic bouton number, pupal lethality [160] Reduced larval crawling, no effect on larval CNS size, 70% of adult eclosion [165] Larval locomotion impaired, pupal lethality [164]
	FUS R495X	Normal larval locomotion, no defect in adult eclosion, no defect in climbing activity, no effect on adult viability [164]
	FUS R518K	Impaired larval locomotion, no effect on synaptic boutons, pupal lethality [158] Drastic larval crawling reduction, reduction in larval CNS size, no adult eclosion [165]
	FUS R521C	Impaired larval locomotion, no effect on synaptic boutons, pupal lethality [158] Drastic larval crawling reduction, reduction in larval CNS size, no adult eclosion [165]
	FUS R521G	Larval locomotion activity impaired, decreased synaptic bouton number, pupal lethality [160]
	FUS R521H	Impaired larval locomotion, no effect on synaptic boutons, pupal lethality [158]
	FUS R524S	Disruption in MNs cluster, decreased synaptic bouton number, reduction in mobility, tail lifted [147]
	FUS P525L	Disruption in MNs cluster, decreased synaptic bouton number, reduction in mobility, tail lifted [147] Larval locomotion impaired, pupal lethality [164]
	FUS 4F-L (RRM Mutant)	No effect on larval crawling, no effect on the larval CNS size, no effect on adult eclosion [165]
	FUSΔ32	No effect on larval locomotion, no effect on synaptic bouton number [160]
OK6-Gal4 (motoneurons)	FUS WT	Increased synaptic bouton number [159] Adult eclosion defect with immature escapers [166]
	FUS R521G	Adult eclosion defect with immature escapers [166]
	FUS R521H	Adult eclosion defect with immature escapers [166]
	FUS R522G	No effect on synaptic bouton number [159]
	FUS P525L	No effect on synaptic bouton number [159]
GMR-Gal4 (eye)	FUS WT	Very mild rough eye [158,165] Reduction in red pigment, rough surface [147] Malformed interommatidial bristles [162] Severe rough eye phenotype [160] Rough eye with pigment loss [164] Reduced and rough eye [167]
	FUS R495X	Mild rough eye with slight pigmentation defect [164]
	FUS R518K	Rough eye [158,165,167]
	FUS R521C	Rough eye [158,160,165,167]
	FUS R521G	Rough eye [160]
	FUS R521H	Rough eye [158]
	FUS R524S	Severe rough eye [147]
	FUS P525L	Severe rough eye, depigmentation [147] Rough eye with pigment loss [164]
	FUSΔ32Cter	No phenotype [160]
	FUS 4F-L (RRM Mutant)	No phenotype [165]
CCAP-Gal4 (bursicon neurons)	FUS WT	Adult eclosion impairment and escapers immature phenotype [166]
	FUS R521G	Adult eclosion impairment and escapers immature phenotype [166]
	FUS R521H	Adult eclosion impairment and escapers immature phenotype [166]
OK107-Gal4 (mushroom bodies)	FUS WT	Thin mushroom body lobes [147]
	FUS R524S	Drastic decreased size of MB neurons, axonal degeneration [147]
	FUS P525L	Drastic decreased size of MB neurons, axonal degeneration [147]
MS1096-Gal4 (wing pouch)	FUS WT	Defect in wing formation [160]
	FUS R521G	Defect in wing formation [160]
	FUS Δ32	No effect [160]

This table describes the phenotypes associated with different UAS FUS lines. Note that the experiments could be done at different temperatures and the UAS lines were generated using different genetic strategies (site-specific or random insertion) and tags.

**Table 4:** Phenotypes observed in *cabeza* mutants.

Mutant Line	Phenotype	Reference
*caz^1^*	No effect on NMJ morphology Adult eclosion impairment Adult locomotion affected Reduced lifespan	[159]
	Loss of ommatidia in the eyes	[174]
*caz^2^*	Developmental delay and pupal lethality	[175]
*caz^KO^*	Developmental delay and pupal lethality	[175]
caz^lox^, elav-Gal4, UAS Cre (pan-neuronal)	Reduced adult offspring Adult motor deficit Reduced lifespan	[175]
caz^lox^, Mef2-Gal4, UAS Cre (muscles)	Reduced muscle width	[175]
Caz^FRT^, elav-Gal4, UAS FLP (pan-neuronal)	Reduced adult offspring Reduced lifespan	[175]
Caz^FRT^, Mef2-Gal4, UAS FLP (muscles)	No effect	[175]

This table describes the phenotypes associated with different *cabeza* mutant lines. Note that the experiments could be done at different temperatures.

**Table 5:** Cabeza gain of function and RNAi-induced *cabeza* silencing phenotypes.

Gal4 Line	Line	Phenotype/Reference
Act5C-Gal4 (ubiquitous)	UAS-caz	Lethal, no adult eclosion [160]
	UAS-RNAi caz (363–399) VDRC 100291	Low adult eclosion [160] No effect at 28 °C [150]
	UAS-RNAi caz (1–167)	Lethal at 28 °C, no effect at 25 °C [150] Late pupal lethality [176]
	UAS-RNAi caz (180–346)	No effect at 28 °C [150]
Elav-Gal4 (pan neuronal)	UAS-caz	Almost lethal at pupal stage, few escapers [160]
	UAS-RNAi caz (363–399) VDRC 100291	Adult eclosion defect [160] No effect on lifespan, reduced mobility from young adult, decreased in total axonal branch length [150] and synaptic bouton number [177] Adult climbing defect, number of synaptic bouton and total branch length reduced [178,179]
	UAS-RNAi caz (1–167)	No effect on lifespan, reduced mobility from young adult. In larvae, decreased synaptic bouton number [150,177] and decreased total axonal branch length [150]
	UAS-RNAi caz (180–346)	No effect on lifespan [150]
D42-Gal4 (motoneurons)	UAS-Caz	Pupal lethality, no adult eclosion [160]
	UAS-RNAi caz (363–399) VDRC 100291	Low adult eclosion [160]
OK6-Gal4 (motoneurons)	UAS-caz	Increased synaptic bouton number [159] Rescued the *caz^1^* phenotypes [159]
OK371-Gal4 (glutamatergic neurons)	UAS-caz	Severe larval crawling defect, reduction of synaptic bouton number [160]
GMR-Gal4 (eye)	UAS-caz	Rough eye [160,177]
	UAS-RNAi caz (1–167)	Rough eye [176,178]
	UAS-RNAi caz (363–399) VDRC 100291	Severe rough eye [179,180] apoptosis [176]
nsyb-Gal4 (pan neuronal)	UAS-RNAi caz (363–399) VDRC 100291	No significant lethality, slight motor defect [175]
	UAS-RNAi HMS00790	No significant lethality, slight motor defect [175]
	UAS-RNAi HMS00156	No significant lethality, slight motor defect [175]
Ppk-Gal4 (da neuron)	UAS-caz	Reduced synaptic projections [181]
	UAS-caz P398L	Reduced synaptic projections [181]

This table describes the phenotypes associated with different UAS-*cabeza* or UAS RNAi *cabeza* lines. Note that the experiments could be done at different temperatures and the UAS lines were generated using different genetic strategies (site-specific or random insertion).

**Table 6:** TDP-43 homolog (*TBPH*) mutants cited in this review. mRNA, messenger RNA.

Mutant Name	Description	*TBPH* mRNA/Protein	Phenotype
*TBPH* Δ*23* [267]	1.6 kb deletion (promoter region + exon 1 and part of exon 2)	Absent protein (WB adult head + Immuno on larval and adult brain) Ab against AAs 1–268	Lethality throughout development; adult escapers with strongly reduced lifespan, incapacity of walking and climbing
*TBPH* Δ*142* [267]	0.8 kb deletion (promoter region)	Absent protein (WB adult head) Ab against AAs 1–268	Lethality throughout development; adult escapers with strongly reduced lifespan, incapacity of walking and climbing
*TBPH Q367X* [274]	Single nucleotide change introducing STOP codon at amino acid 367	Absent protein (WB adult head) Ab against AAs 179–192	Semi-lethal with adult escapers
*TBPH KO* [268]	Deletion of entire coding sequence	Absent mRNA (qRT-PCR on first instar larval CNS)	Lethal at 2nd instar larval stage
*TBPH ex26* [265]	932 bp deletion (promoter region)	Absent mRNA (RT-PCR all developmental stages) Absent protein (Immuno on larval brain and muscle) Ab against AAs 307–531	Semi-lethal with adult escapers having severe movement defects
*TBPH G2* [275]	Deletion (promoter region + part of exon 1)	Absent protein (WB third instar larval CNS) Ab against full length protein	100% pupal lethal
*TBPH DD100* [266]	Deletion (promoter region + part of exon 1)	Absent protein (WB adult head + Immuno on adult brain) Ab against AAs 291–305 or 517–531	Lethality throughout development, adult escapers with strongly reduced lifespan and impaired climbing
*TBPH DD96* [266]	Deletion (promoter region + exons 1–3 + part of exon 4)	Absent protein (WB adult head) Ab against AAs 291–305 or 517–531	Lethality throughout development, adult escapers with strongly reduced lifespan and impaired climbing

This table describes the name and the associated reference of each mutant, the type of mutation, the detection of *TBPH* RNA and/or protein, and the major phenotypes of each mutant (AAs: amino acids; Ab: antibody; Immuno: immunohistochemistry; WB: Western blot).

**Table 7:** Phenotypes at larval neuromuscular junction of *TBPH* mutant.

Mutant	Synaptic Boutons Number	Axonal Branch Number	Axonal Branch Length	Active Zone Number	Larval Locomotion Defect
*TBPH* Δ23/Δ23	Reduced [267,280] Normal [281]	Reduced [267,280]	Normal [281]	nd	Yes [267]
*TBPH*Δ23/Df[2R]BSC610	Normal [281]	nd	Normal [281]	nd	nd
*TBPH* Δ23/Df[2R]BSC660	Normal [159]	Normal [159]	Normal [159]	nd	Yes [159]
*TBPH* Δ142 Δ142	Reduced [267]	Reduced [267]	nd	nd	Yes [267]
*TBPH* ex26/ex26	Increased [265]	Increased [265]	nd	nd	Yes [265]
*TBPH* ^G2/G2^	Normal [277]	Normal [277]	Normal [277]	nd	Yes [277]
*TBPH* DD100/DD100	Normal [266]	nd	nd	Normal [266]	Yes [266]
*TBPH* DD96/DD96	Normal [266]	nd	nd	Normal [266]	Yes [266]

This table describes the *TBPH* mutant conditions and the parameters used for larval neuromuscular junction analyzes (nd: not determined).

**Table 8:** Major phenotypes associated with RNA interference (RNAi)-induced *TPBH* silencing.

Gal4 Line	RNAi Line	Phenotype/Reference
Act5C-Gal4 (ubiquitous)	104401 (VDRC)	Lethal [269]
	38377 (VDRC)	Lethal [269] Adult flies with ectopic bristles [287]
Tubulin-Gal4 (ubiquitous)	38377 (VDRC)	Lethal pupal with adult escapers [274]
	38379 (VDRC)	Lethal pupal with adult escapers [274]
	Homemade RNAi targeting exon 5	Larval/pupal lethality with adult escapers, impaired adult motor behavior [266]
Tubulin-Gal4, Tubulin-Gal80TS (ubiquitous, induced at adult stage)	38379 (VDRC)	Climbing defect, reduced lifespan [288]
Elav-Gal4 (pan-neuronal)	38377 (VDRC)	Impaired larval locomotion [265] Age progressive climbing deficit [267,269] Reduced lifespan [269]
	38379 (VDRC)	Age progressive climbing deficit [267]
	104401 (VDRC)	Age progressive climbing deficit, reduced lifespan [269]
	29517 (BDSC)	Age progressive climbing deficit [269]
	39014 (BDSC)	Age progressive climbing deficit [269]
	Homemade RNAi targeting exon 5	Impaired synaptic transmission (increase quantal content) at larval NMJ and abnormal adult motor behavior [266]
Elav-Gal4, Tubulin-Gal80TS (pan-neuronal, induced at larval stage)	38379 (VDRC)	Abnormal distribution of Dlg and GluRIIA at larval NMJs [288]
1407-Gal4 (pan-neuronal)	38377 (VDRC)	Strong climbing defect [267]
D42-Gal4 (motoneurons)	38377 (VDRC)	Normal larval locomotion [265] Increased larval turning, age progressive climbing deficit [289]
	38379 (VDRC)	Increased larval turning, age progressive climbing deficit [289]
OK371-Gal4 (glutamatergic neurons)	38377 (VDRC)	Normal larval locomotion [290]
OK371-Gal4/MARCM (individual leg motoneuron)	38379 (VDRC)	Normal larval locomotion [290]
	Unknown	No effect on leg motoneuron/axon [291]
Repo-Gal4 (pan-glial)	38379 (VDRC)	Larval locomotion and climbing defects, reduced lifespan, wrapping defect at larval NMJ [271]
	Homemade RNAi targeting exon 5	Normal lifespan and climbing, impaired overall motor activity in 30 day old flies [270]
BG57-Gal4 (muscles)	Homemade RNAi targeting exon 5	Impaired synaptic transmission at larval NMJ (normal quantal content) [266]
Mef2-Gal4 (muscles)	38379 (VDRC)	Impaired larval locomotion and climbing, reduced lifespan [282]
	Homemade RNAi targeting exon 5	Normal lifespan and climbing, impaired overall motor activity in 30 day old flies [270]
Mhc-Gal4 (muscles)	38377 (VDRC)	Normal larval locomotion [265] Impaired larval locomotion and climbing [282]
GMR-Gal4 (eye)	38377 (VDRC)	No effect [290] Mild rough eye [269,292]
	38379 (VDRC)	No effect [290]
	104401 (VDRC)	Mild rough eye [269]
	29517 (BDSC)	Mild rough eye [269]
	39014 (BDSC)	Mild rough eye [269]
	Unknown	No effect [272], weak eye degeneration [293]
EB1-Gal4 (ellipsoid body neurons)	Homemade RNAi targeting exon 5	Impaired adult motor behavior, age dependent axonal/synaptic degeneration and loss of upper motoneuron [266]
Gal4^221^-Gal4 (sensory neurons)	38377 (VDRC)	Reduced dendritic branching [274]
	38379 (VDRC)	Reduced dendritic branching [274]
OK107-Gal4 (mushroom bodies)	38377 (VDRC)	Normal mushroom bodies, weak learning inability [265] Axonal loss and neuronal death [290]
	38379 (VDRC)	Normal mushroom bodies and learning ability [265] Axonal loss and neuronal death [290]

This table describes the Gal4 lines used to express *TBPH* RNAi, the type/origin of RNAi construct, and the associated phenotypes/references. It is important to note that all experiments were not done at the same temperature. In addition, some experiments have combined RNAi and Dicer-2 expression to enhance the effect of the RNAi (not mentioned). Altogether, these different experimental conditions may explain some discrepancies. Data obtained at larval NMJs are not described.

**Table 9:** Gain-of-function phenotypes induced by *TBPH.*

Gal4 Line	Phenotype/Reference
Elav-Gal4 (pan-neuronal)	100% lethality (90% at first instar larvae) [275] Semi-lethal [274] Lethal throughout development, larval locomotion defect, adult escapers with reduced lifespan and impaired climbing [266]
Elav-Gal4, Tubulin-Gal80TS (pan-neuronal, induced at adult stage)	Climbing defect [288]
D42-Gal4 (motoneurons)	Impaired larval locomotion [265] Increased larval turning, weak motoneuronal death in LIII ventral ganglia [289] Lethal pupal stage [265,289] Lethal pupal/reduced lifespan depending on the dose [265] Age-dependent climbing defect [265,275]
D42-Gal4, Tubulin-Gal80TS (motoneurons, induced at adult stage)	Weak reduction of neuron number in thoracic ganglia, reduced lifespan [301]
OK371-Gal4 (glutamatergic neurons)	Lifespan severely reduced [302]
OK371-Gal4/MARCM (individual leg motoneuron)	Leg motor axon and NMJ degeneration [291]
Repo-Gal4 (pan-glial)	Premature lethality (prepupal stage) [270]
Mef2-Gal4 (muscles)	Irregular sarcomere (2nd instar larval stage), impaired larval locomotion, prepupal lethality [270]
GMR-Gal4 (eye)	Eye degeneration [270,274,289,292,302,303,304] 100% lethal at 29 °C [289]
EB1-Gal4 (ellipsoid body neurons)	Impaired adult motor behavior, age-dependent axonal/synaptic degeneration, and loss of upper motoneuron [266]
Gal4^221^-Gal4 (sensory neurons)	Increased dendritic branching [274]
OK107-Gal4 (mushroom bodies)	Smaller axonal lobe, impaired learning ability [265]

This table describes the Gal4 lines used to overexpress *TBPH* and the associated phenotypes/references.

**Table 10:** Gain-of-function phenotypes induced by wildtype TDP-43.

Gal4 Line	Phenotype/Reference
Act5C-Gal4 (ubiquitous)	Premature lethality [305]
Daughterless-Gal4 (ubiquitous)	Lethal pupal [306]
Elav-Gal4 (pan-neuronal)	Embryonic lethal [306] L1 lethal [307] Reduced lifespan [305,308,309,310] Age progressive climbing deficit [309,311]
Elav-Gal4, Tubulin-Gal80TS (pan-neuronal, induced at adult stage)	Premature lethality, reduced lifespan [305,307] Age progressive climbing deficit [312]
ElavGS (pan-neuronal, induced at adult stage)	Reduced lifespan [307,313] Age progressive climbing deficit [314]
D42-Gal4 (motoneurons)	Reduced survival, lethal at 2nd instar larval stage with high dose [315] Semi-lethal at 25 °C but viable at 18 °C, impaired climbing, no motoneuronal death in LIII ventral ganglia, motoneuronal death in adult thoracic ganglia [289] Larval locomotion defect [316] Increased larval turning [289,291,317,318,319] Age progressive climbing deficit [305,308,316,320,321] Reduced lifespan [293,313,318,320,322,323]
D42-Gal4, Tubulin- Gal80TS (motoneurons, induced at adult stage)	Age progressive climbing deficit, reduced lifespan [324]
OK371-Gal4 (glutamatergic neurons)	Lethal [306] Lethal pupal, motoneuron death in LIII [290]
OK371-Gal4/MARCM (individual leg motoneuron)	Leg motor axon and NMJ degeneration [291]
RN2-Gal4 (sub-population of adult motoneurons)	Age progressive climbing deficit [290]
Repo-Gal4 (pan-glial)	Pupal lethal [307] Age progressive climbing deficit, reduced lifespan [309,325] Terminal deoxynucleotidyl tranferase (TdT) dUTP Nick-end labeling (TUNEL)-positive neural cells [309] Little loss of glial cells and neurons in adult brains [326]
Repo-Gal4, Tubulin-Gal80TS (pan-glial, induced at adult stage)	Dramatic loss of glial cells associated to neuronal loss in adult brains [326]
24B-Gal4 (muscles)	Lethal [306,307]
Mef2-Gal4 (muscles)	Lethal [306]
GMR-Gal4 (eye)	Neurodegeneration [310,312,316,317,319,320,324,327,328,329] Age dependent progressive neurodegeneration [289,290,293,307,308,314,315,318,330,331,332,333,334]
GMR-Gal4, Tubulin-Gal80TS (eye, induced at adult stage)	Age-dependent progressive neurodegeneration [293,312]
Gal4^221^-Gal4 (sensory neurons)	Promoted dendritic branching [274]
OK107-Gal4 (mushroom bodies)	No TUNEL-positive cells [309] Axonal loss and neuronal death (TUNEL-positive cells) [290]

This table describes the Gal4 lines used to overexpress TDP43 and the associated phenotypes/references.

**Table 11:** Gain-of-function phenotypes induced by *TBPH* or TDP-43 at larval neuromuscular junctions.

Gal4	*TBPH* or TDP-43	Active Zone Number	Synaptic Boutons Number	Axonal Branch Number	Axonal Branch Length	Larval Locomotion
Elav-Gal4 (pan-neuronal)	TDP-43	nd	Decreased [316]	nd	nd	nd
D42-Gal4 (motoneurons)	*TBPH*	nd	Increased [289] Decreased, dose dependent [265]	Normal [289] Decreased, dose dependent [265]	nd	Altered larval turning [289] Impaired, dose dependent [265] Normal [275]
	TDP-43	Increased [289]	Decreased [11,289,335,336] Normal [293]	Decreased [11,289,335]	nd	Altered larval turning [289] Normal [293]
OK6-Gal4 (motoneurons)	*TBPH*	nd	Increased [159]	nd	Increased [159]	nd
	TDP-43	nd	Increased [159]	nd	Increased [159]	nd
OK371-Gal4 (glutamatergic neurons)	*TBPH*	nd	Normal [302]	nd	nd	nd
	TDP-43	Normal [337]	Decreased [281,290]	Decreased [143]	Decreased [281]	Altered [281,290]
Repo-Gal4 (pan-glial)	TDP-43	Normal [336]	Decreased [336]	nd	nd	Altered larval turning [336]

This table describes the Gal4 lines used to overexpress *TBPH* or TDP-43 and the associated phenotypes/references at larval neuromuscular junctions (nd: not determined).

**Table 12:** Comparison of phenotypes induced by gain of function of TDP-43 wildtype, mutated in the NLS or in the NES.

GAL4 Line	TDP-43^Ω^	TDP-43^NLSmut^	TDP-43^NESmut^
Act5C-Gal4 (ubiquitous)	Lethal [305]	Lethal [305]	nd
Elav-Gal4 (pan-neuronal)	L1 larval lethality [307] Viable, strong reduced life span [305]	Pupal lethal [307] Viable, modest reduced life span [305]	Pupal lethal, rare escapers [307]
ElavGS (pan-neuronal, induced at adult stage)	Strong reduced lifespan [307]	Modest reduced lifespan [307]	Weak reduced lifespan [307]
D42-Gal4 (motoneurons)	Age progressive climbing defect [321]	Strong age progressive climbing defect [321]	No climbing defect [321]
Repo-Gal4 (pan-glial)	Pupal lethal [307]	Pupal lethal [307]	No effect [307]
24B-Gal4 (muscles)	Larval lethal [307]	Embryonic lethal [307]	No effect [307]
GMR-Gal4 (eye)	Modest degeneration [292,293,307,321,327]	Strong degeneration [292,293,307,321,327]	No degeneration [292,307,321]

This table describes the Gal4 lines used to overexpress the wildtype or NLS/NES mutant form of TDP-43 and the associated phenotypes/references.

**Table 13:** Gain-of-function phenotypes induced by ALS-linked TDP-43 mutants.

TDP-43 Mutant	Gal4 Line	Phenotype/Reference
D169G	D42-Gal4 (motoneurons)	Decreased bouton number [336] Increased active zone number [336] Increased larval turning time [331,336] Abnormal nuclear shape [336]
	Repo-Gal4 (pan-glial)	Normal bouton number (reduced with WT) [336] Normal active zone number [336] Increased GluRIIC [336] Increased larval turning time [336]
	GMR-Gal4 (eye)	Age and dose dependent degeneration [331,336]
G287S	Act5C-Gal4 (ubiquitous)	Lethal [305]
	Elav-Gal4 (pan-neuronal)	Viable, reduced lifespan (as WT but better) [305]
G298S	Elav-Gal4 (pan-neuronal)	Impaired climbing [311]
	D42-Gal4 (motoneurons)	Decreased bouton number [336], (normal with WT) [293] Increased active zone number [336] Abnormal nuclear shape [336] Increased larval turning time [317,318,319,331,336,340], (normal with WT) [293] Decreased larval locomotion (Normal with WT) [293] Reduced lifespan [318], (as WT but worse) [293]
	Repo-Gal4 (pan-glial)	Decreased bouton number [336] Normal number of active zones [336] Increased GluRIIC [336] Increased larval turning time [336]
	GMR-Gal4 (eye)	Degeneration [317,318,319] Age- and dose-dependent degeneration [336] Age-dependent degeneration (as WT but worse) [293]
A315T	Act5C-Gal4 (ubiquitous)	Lethal [305]
	Elav-Gal4 (pan-neuronal)	Viable, reduced lifespan (as WT but better) [305]
	Elav-Gal4, Tubulin-Gal80TS (pan-neuronal, induced at adult stage)	Age-dependent climbing deficit (as WT but worse) [312]
	D42-Gal4 (motoneurons)	No change in bouton number (WT decreased) [289], decreased at high dose [289,336] No change in branch number, even at high dose (WT decreased) [289] Increased larval turning time (as WT but worse) [289] Increased active zone number [336] Abnormal nuclear shape [336] Viable but 100% pupal lethal at high dose (WT semi-lethal, escapers) [289] Reduced lifespan (as WT but better) [289] Impaired climbing (as WT but better) [289] No motoneuron apoptosis in LIII ganglia [289] Rare motoneuron apoptosis in adult thoracic ganglia (as WT but better) [289] Age progressive climbing deficit [305] Upregulation of *HDAC6* mRNA (as WT but more) [340]
	OK371-Gal4 (glutamatergic neurons)	Cell soma and proximal axon (WT reaches distal axon and NMJ) [296] Impaired anterograde movement (compared to WT) [296]
	Repo-Gal4 (pan-glial)	Normal number of active zones [336] Increased GluRIIC [336]
	GMR-Gal4 (eye)	Age- and dose-dependent degeneration [289,331], (as WT but worse) [293] Degeneration with mitochondrial damage [312]
	GMR-Gal4, Tubulin-Gal80TS (eye, induced at adult stage)	Age-dependent degeneration with mitochondrial damage [312]
Q331K	Daughterless-Gal4 (ubiquitous)	Lethal pupal [306]
	Elav-Gal4 (pan-neuronal)	Embryonic lethal [306]
	D42-Gal4 (motoneurons)	Age progressive climbing deficit (as WT but worse) [308]
	OK371-Gal4 (glutamatergic neurons)	Lethal [306]
	OK371-Gal4/MARCM (individual leg motoneuron)	Degeneration of leg motor axon and NMJ (as WT but worse) [291]
	24B-Gal4 or Mef2-Gal4 (muscles)	Lethal [306]
	GMR-Gal4 (eye)	Cytoplasmic puncta (not observed with WT) [306]
	Gal4^221^-Gal4 (sensory neurons)	Increased dendritic branching (as WT but less) [274]
	OK107-Gal4 (mushroom bodies)	Lethal (WT is viable) [306]
	TH-Gal4 (dopaminergic neurons)	Viable [306]
	Vestigial-Gal4 (wing imaginal discs)	Ectopic production of scutellar bristle (as WT but less) [287]
M337V	Armadillo-Gal4 (ubiquitous)	No rescue of viability of *TBPH* mutant (rescue with WT) [296]
	Daughterless-Gal4 (ubiquitous)	Lethal pupal [306]
	Elav-Gal4(pan-neuronal)	Embryonic lethal [306]
	D42-Gal4 (motoneurons)	Normal bouton number [293] Increased larval turning time (normal with WT) [293] Decreased larval locomotion (normal with WT) [293] Reduced lifespan (as WT but worse) [293]
	OK371-Gal4 (glutamatergic neurons)	Cell soma and proximal axon (WT reaches distal axon and NMJ) [296] Impaired anterograde movement (compared to WT) [296] Lethal [306]
	OK6-Gal4 (motoneurons)	Normal bouton number (increased with WT) [159]
	24B-Gal4 or Mef2-Gal4 (muscles)	Lethal [306]
	GMR-Gal4 (eye)	Age dependent degeneration (as WT but worse) [293,334]
	CCAP-Gal4 (bursicon neurons)	Impaired vesicle transport (no effect with WT) [341]
	Gal4^221^-Gal4 (sensory neurons)	Increased dendritic branching (as WT but less) [274]
	OK107-Gal4 (mushroom bodies)	Viable [306]
	TH-Gal4 (dopaminergic neurons)	Viable [306]
	Vestigial-Gal4 (wing imaginal disc)	Ectopic production of scutellar bristle (as WT but less) [287]
Q343R	GMR-Gal4 (eye)	Age-dependent degeneration (as WT but worse) [293]
N345K	D42-Gal4 (motoneurons)	Decreased bouton number [336] Increased active zone number [336] Increased larval turning time [336] Abnormal nuclear shape [336]
	Repo-Gal4 (pan-glial)	Decreased bouton number [336] Normal number of active zones [336] Increased GluRIIC [336] Increased larval turning time [336]
	GMR-Gal4 (eye)	Age- and dose-dependent degeneration [336]
G348C	Act5C-Gal4 (ubiquitous)	Lethal [305]
	Elav-Gal4 (pan-neuronal)	Viable, reduced lifespan (as WT but better) [305]
A382T	Act5C-Gal4 (ubiquitous)	Lethal [305]
	Elav-Gal4 (pan-neuronal)	Viable, reduced lifespan (as WT but better) [305]
	D42-Gal4 (motoneurons)	Upregulation of *HDAC6* mRNA (as WT but more) [340]
N390D	Act5C-Gal4 (ubiquitous)	Lethal [305]
	Elav-Gal4 (pan-neuronal)	Viable, reduced lifespan (as WT but better) [305]

Most of the phenotypes induced by ALS-linked TDP-43 mutants are similar to those induced by wildtype except when mentioned (text in bold). Note that studies that used ALS-linked mutants without comparing them to the wildtype are not mentioned.

## Data Availability

Not applicable.

## Abbreviations

ALS	Amyotrophic lateral sclerosis
arcRNA	Architectural RNA
Arg	Arginine
ATP	Adenosine triphosphate
Aub	Aubergine
BMAA	β-*N*-methylamino l-alanine
C9ORF72	Chromosome 9 open reading frame 72
Ca	Calcium
Cas9	CRISPR-associated protein 9
Caz	Cabeza
CCAP	Crustacean cardioactive peptide
Chip	Chromatin immunoprecipitation
CNS	Central nervous system
CRISPR	Clustered regularly interspaced short palindromic repeats
Cu	Copper
DART5	*Drosophila* arginine methyltransferase protein 5
dDSIF	*Drosophila* DRB sensitivity-inducing factor
dEAAT1	*Drosophila* excitatory amino acid transporter 1
dFMR1	*Drosophila* Fragile X mental retardation 1
dFMRP	*Drosophila* Fragile X mental retardation protein
Dlg	Discs-large
DNA	Deoxyribonucleic acid
dPAF1	*Drosophila* polymerase-associated factor 1
*DPR*	*Dipeptide proteins*
eIF1A	Eukaryotic translation initiation factor 1A
eIF2α	Eukaryotic initiation factor 2α
EJP	Excitatory junction potential
Elav	Embryonic lethal abnormal visual protein
ER	Endoplasmic reticulum
EWS	Ewing sarcoma
fALS	Familial amyotrophic lateral sclerosis
FET	FUS EWS TAF15 family of proteins
FUS	Fused in sarcoma
FTD	Frontotemporal dementia
Gad1	Glutamic acid decarboxylase 1
GDP	Guanosine diphosphate
GFP	Green fluorescent protein
GluRIIA	Glutamate receptor IIA
Gly	Glycine
GMR	Glass multiple reporter
GOF	Gain of function
GRD	Glycine-rich domain
GS	GeneSwitch
GTP	Guanosine triphosphate
HDAC6	Histone deacetylase 6
HEK/HEK293	Human embryonic kidney 293
HeLa	Henrietta Lacks
Hpo	Hippo
hnRNP	Heterogenous nuclear ribonucleoprotein
HSP60	Heat-shock protein 60
HSP70	Heat-shock protein 70
HRE	Hexanucleotide repeat expansion
IP3	Inositol 1,4,5-trisphosphate
iPSC	Induced pluripotent stem cell
ITPR1	Inositol-1,4,5-trisphosphate receptor type 1
JNK	c-Jun N-terminal kinase
Kapβ2	Karyopherin-β2
kDa	Kilodalton
mTORC1	Mammalian target of rapamycin complex 1
LA	α-Lipoic acid
LAMP-1	Lysosomal-associated membrane protein 1
LCD	Low-complexity domain
lncRNA	Long non-coding RNA
LOF	Loss of function
MARCM	Mosaic analysis with a repressible cell marker
miRNA	MicroRNAs
mRNA	Messenger RNA
NB	Nuclear body
NES	Nuclear export signal
NIR	Nuclear import receptor
NMJ	Neuromuscular junction
NLS	Nuclear localization signal
Nup	Nucleoporin
Orz	γ-Oryzanol
PABPN1	Poly(A)-binding protein nuclear 1
PARG	Poly(ADP-ribose) glycohydrolase
PARP1	Poly(ADP-ribose)ylation polymerase 1
PEK	Poly(ADP-ribose)ylation glycohydrolase
PINK1	PTEN-induced kinase 1
piRNA	Piwi-interacting RNA
PrLD	Prion-like domain
poly-GA	Poly-glycine/alanine
poly-GP	Poly-glycine/proline
poly-GR	Poly-glycine/arginine
poly-PA	Poly-proline/alanine
poly-PR	Poly-proline/arginine
PTM	Post-translational modification
PTP1B	Tyrosine phosphatase 1B
qPCR	Quantitative polymerase chain reaction
QGSY	Glutamine, glycine, serine, and tyrosine residues
RAN	Repeat-associated non-AUG
RanGAP	Ras-related nuclear GTPase-activating protein
RAVER1	Ribonucleoprotein, PTB-binding 1
RBP	RNA-binding protein
Rbp1	RNA-binding protein 1
RGG	Arginine glycine rich domain
ROS	Reactive oxygen species
RRM	RNA recognition motif
RNA	Ribonucleic acid
RNAi	RNA interference
RTE	Retrotransposable element
RT-PCR	Reverse transcriptase polymerase chain reaction
sALS	Sporadic amyotrophic lateral sclerosis
SF2	Splicing factor 2
Sf3b-1	Splicing factor 3b subunit 1
SG	Stress granule
siRNA	Small interfering RNA
SOD	Superoxide dismutase
SOP	Sensory organ precursor
TAF15	TATA box-binding protein-associated factor 68 kDa
TARDBP	TAR DNA-binding protein
TARGET	Temporal and regional gene expression targeting
*TBPH*	TAR DNA-binding protein-43 homolog
TCERG1	Transcription elongation regulator 1
TDP-43	TAR DNA-binding protein 43
TDPBR	TDP-43 binding region
Ter94	Transitional endoplasmic reticulum 94
TLS	Translocated in liposarcoma
TMPyP4	5,10,15,20-Tetrakis-(*N*-methyl-4-pyridyl)porphine
UAS	Upstream activating sequence
UBPY	Ubiquitin isopeptidase Y
UPR	Unfolded protein response
UTR	Untranslated transcribed region
VCP	Valosin-containing protein
Wnd	Wallenda
WT	Wildtype
XPO1	Exportin 1
Zfp106	Zing finger protein 106
Zn	Zinc
ZnF	Zinc finger domain
